# Photocatalytic trifluoromethoxylation of arenes and heteroarenes in continuous-flow

**DOI:** 10.3762/bjoc.16.111

**Published:** 2020-06-15

**Authors:** Alexander V Nyuchev, Ting Wan, Borja Cendón, Carlo Sambiagio, Job J C Struijs, Michelle Ho, Moisés Gulías, Ying Wang, Timothy Noël

**Affiliations:** 1 Micro Flow Chemistry and Synthetic Methodology, Department of Chemical Engineering and Chemistry, Eindhoven University of Technology, Het Kranenveld, Bldg 14 − Helix, 5600 MB Eindhoven, The Netherlands; 2 Centro Singular de Investigación en Química Biolóxica e Materiais Moleculares (CIQUS) and Departamento de Química Orgánica, Universidade de Santiago de Compostela, 15782 Santiago de Compostela, Spain; 3 Discovery Chemistry and Technologies, AbbVie Inc., 1 North Waukegan, Road, North Chicago, Illinois 60064, United States of America

**Keywords:** C–H functionalization, continuous-flow, organic synthesis, photoredox catalysis, trifluoromethoxylation

## Abstract

The first example of photocatalytic trifluoromethoxylation of arenes and heteroarenes under continuous-flow conditions is described. Application of continuous-flow microreactor technology allowed to reduce the residence time up to 16 times in comparison to the batch procedure, while achieving similar or higher yields. In addition, the use of inorganic bases was demonstrated to increase the reaction yield under batch conditions.

## Introduction

**Scheme 1:**
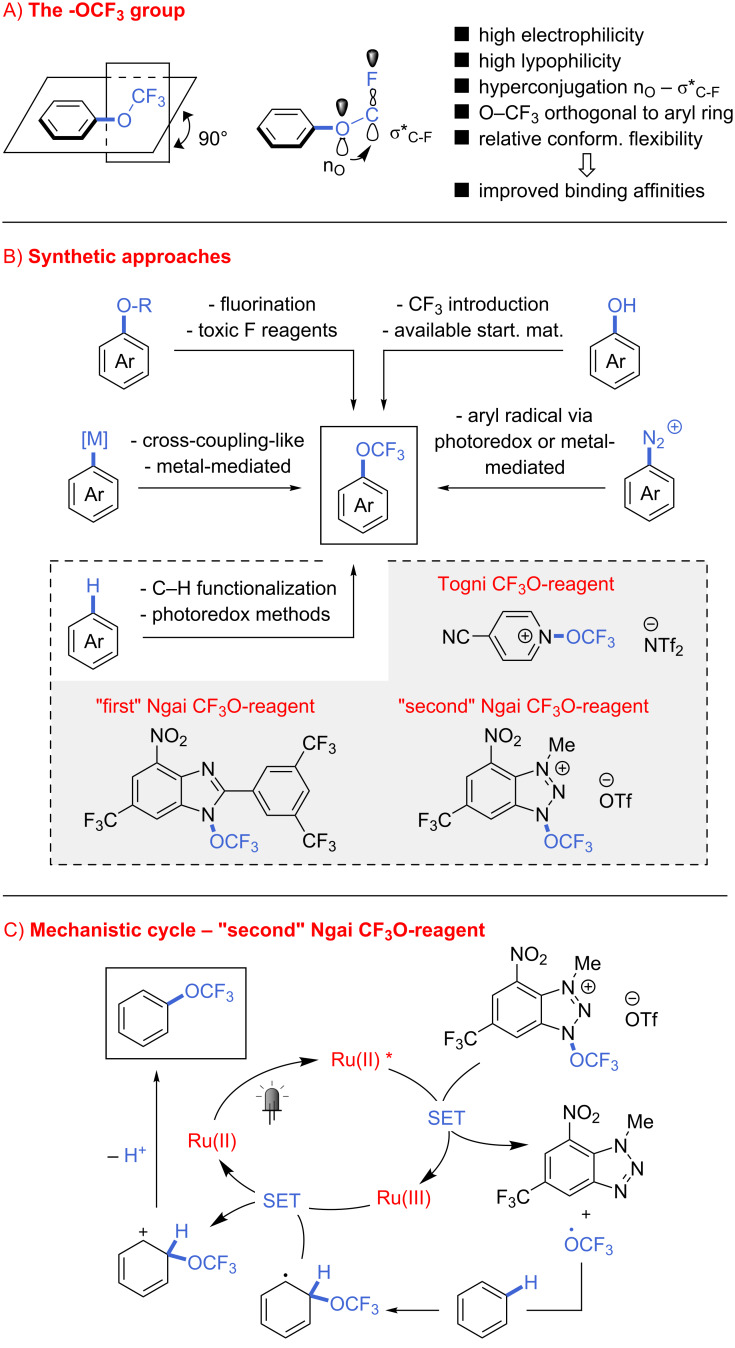
A) Properties and B) synthesis of CF_3_O-bearing arenes; C) trifluoromethoxylation using the “second” Ngai reagent.

The number of fluorine-containing compounds and their influence in medicine and agrochemistry are increasing every year [1–2]. The introduction in a drug (or a bioactive compound in general) of single fluorine atoms, or multiple fluorine-containing substituents, such as -CF_3_, -SCF_3_, -CF_2_H/-CF_2_-, has a range of effects on the pharmacokinetics and pharmacodynamics of the molecule [3]. Among other fluorine-based substituents, the trifluoromethoxy group (-OCF_3_) has until recently remained less explored and understood [4]. Nonetheless, it possesses unique properties, for example, a high electronegativity (χ = 3.7), and a high lipophilicity (Hansch-Leo parameter π = 1.04, intermediate between the -CF_3_ and the -SCF_3_ values, respectively, 0.88 and 1.44) [4]. From a pharmaceutical perspective, the former property provides stability of the drug under physiological conditions, and the latter ensures better solubility and transport across the physiological lipophilic environment (e.g., dissolution in lymph and cell membrane penetration) [5]. In trifluoromethoxylated aryls, the -OCF_3_ group assumes a peculiar orthogonal conformation relative to the aromatic ring, partially due to the hyperconjugation of an oxygen lone pair with an antibonding C–F orbital, and partially to the steric hindrance of the -CF_3_ group [6–7]. These interactions result in a relative conformational flexibility, where the -OCF_3_ group can rotate between the two sides of the aromatic ring (Scheme 1A). This property might be responsible for stronger binding affinities of trifluoromethoxylated compounds with the active sites in enzymes, proteins, or other biomolecules [8–9].

Several procedures for the synthesis of trifluoromethyl aryl ethers were reported from the mid-1900s, mostly based on the transformation of pre-functionalized aryls, such as trichloromethyl ethers, fluoroformates, or carbonochloridothionate derivatives. These procedures typically required harsh conditions and the use of aggressive and toxic chemicals like BF_3_, HF, MoF_6_, SF_4_, and SbF_3_ [10]. Alternative approaches were recently reported [8–9,11], for example the trifluoromethylation of hydroxyaryls [12–14], and the direct introduction of the -OCF_3_ moiety into an organometallic species [15], a diazo compound [16–17], or an unfunctionalized C–H bond (Scheme 1B). The latter method is particularly interesting, partially due to the general interest of the community into C–H functionalization methodologies [18–19], and partially thanks to the synthetic possibilities enabled by photoredox catalysis, a field widely explored in recent years [20–22]. In 2018, Ngai [23–24] and Togni [25] reported simple photoredox protocols for the radical trifluoromethoxylation of unfunctionalized (hetero)arenes by using specifically designed CF_3_O radical-releasing agents (Scheme 1B).

Following our long-standing interest in the development of continuous-flow approaches for C–H functionalization [18,26] and photochemical methodologies [27–31], herein we report the first application of flow methods for the direct trifluoromethoxylation of arenes and heteroarenes.

## Results and Discussion

Among the three reagents shown in Scheme 1B, we decided to use the “second” Ngai reagent (**1**, Scheme 1C) for our investigation, as less side-products (derived from alternative radical formation from the other two reagents [23,25]) and tolerance towards water and air gives this reagent a certain advantage over the others [24]. To transfer the batch procedure into continuous-flow conditions, the concentration of **1** was initially decreased from the original 0.2 M to 0.04 M, and the amount of substrate increased from 10 to 25 equivalents, while the same catalyst ([Ru(bpy)_3_](PF_6_)_2_) and reaction medium (CH_3_CN/CH_2_Cl_2_ 1:1) were used. Upon irradiation with blue light (465 nm, 18 W LED), the desired trifluoromethoxybenzene (**2**) was obtained in 40% yield after a residence time of 1 h (Table 1, entry 1). A 400 nm LED light led to a higher yield (52%, Table 1, entry 2), thus this light source was used for further investigations. As CH_2_Cl_2_ is not a recommended solvent, especially for industrial and pharmaceutical applications [32], we tested the reaction in pure acetonitrile. As the same yield was obtained (Table 1, entry 3), this solvent was chosen as reaction medium for the following experiments. Either doubling the amount of catalyst or reducing the concentration resulted in considerably higher reaction yields (68 and 65%, Table 1, entries 4 and 5). Increased catalyst loading at 0.08 M produced no improvement (Table 1, entry 6). We next tested the reaction using a lower excess of substrate (10 equiv), obtaining product **2** in 57% yield (Table 1, entry 7). Little improvement was seen upon degassing the reaction mixture prior to the reaction (three freeze-pump-thaw cycles, 63%, Table 1, entry 8). Performing the reaction at 0.2 M concentration gave 61% yield (Table 1, entry 9). Last, we were particularly pleased to see that a sensible increase in yield could be obtained by using a freshly prepared reagent **1** in the reaction (the activity of reagent **1** persists at a primary level during near two weeks when stored in the dark at −20 °C). A yield of 73% could be achieved at 0.08 M, without the tedious degassing procedure (Table 1, entry 10).

**Table 1:** Reaction optimization under flow conditions.

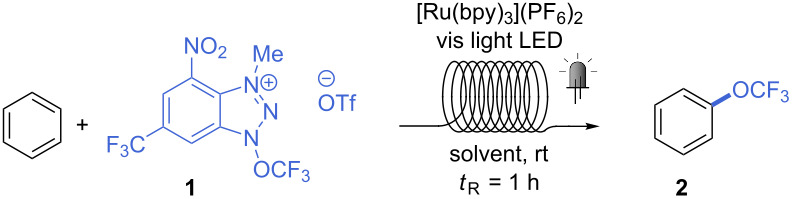					

Entry	Equiv of substrate	Solvent, conc. of **1** (M)	Light source	Cat. (%)	Yield (%)^a^

1	25	CH_3_CN/CH_2_Cl, 0.04	465 nm, 18 W	1	40
2	25	CH_3_CN/CH_2_Cl, 0.04	400 nm, 10 W	1	52
3	25	CH_3_CN, 0.04	400 nm, 10 W	1	53
4	25	CH_3_CN, 0.04	400 nm, 10 W	2	68
5	25	CH_3_CN, 0.08	400 nm, 10 W	1	65
6	25	CH_3_CN, 0.08	400 nm, 10 W	2	64
7	10	CH_3_CN, 0.08	400 nm, 10 W	1	57
8	10	CH_3_CN, 0.08	400 nm, 10 W	1	63^b^
9	10	CH_3_CN, 0.2	400 nm, 10 W	1	61
**10**	**10**	**CH_3_CN, 0.08**	**400 nm, 10 W**	**1**	**73^c^**

control experiments					

11	25	CH_3_CN, 0.04	465 nm, 18 W	1	10^d^
12	25	CH_3_CN, 0.04	400 nm, 10 W	0	17
13	25	CH_3_CN, 0.04	465 nm, 18 W	0	0

^a 19^F NMR yields are reported; ^b^ Degassed solvent; ^c^ Freshly prepared; ^d^ [Ir(dtbbpy)(ppy)_2_]PF_6_ as catalyst.
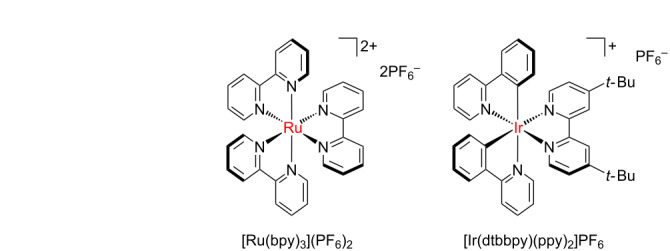

Finally, a few control experiments were performed (Table 1, entries 11–13). A more expensive Ir photocatalyst was tested under blue light irradiation, but a poor 10% yield was obtained. Experiments in the absence of photocatalyst under violet or blue light gave respectively 17% and 0% yield. The little product obtained with 400 nm light can be explained by the photodecomposition of **1**, which generates small amounts of CF_3_O radical (this has been shown for the “first” Ngai reagent [23]).

**Scheme 2:**
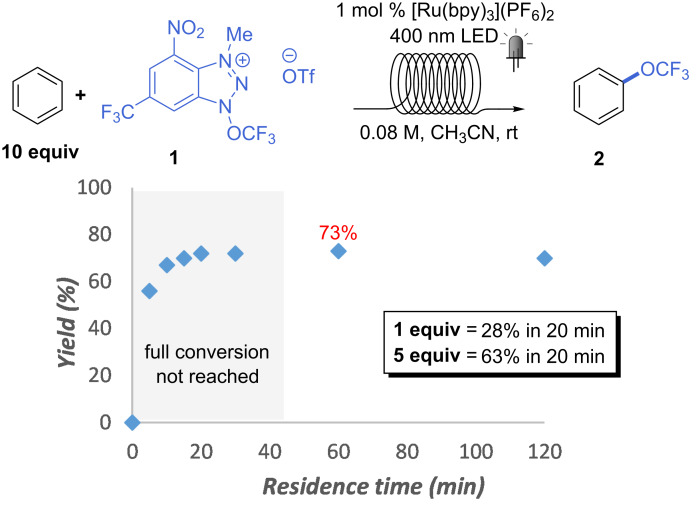
Optimization of residence time. ^19^F NMR yields are reported.

Under the conditions shown in Table 1, entry 10, we then set to figure out the optimal residence time (*t*_R_) for the reaction (Scheme 2). Yields of around 70% were observed for the trifluoromethoxylation of benzene even at 20 minutes residence time, which demonstrates the effectiveness of the flow process (a yield of 63% was obtained in batch over 16 h [24]). However, unreacted **1** was observed at this residence time, and full conversion could be obtained only after 1 h. Moreover, a residence time of 20 minutes proved not sufficient for several other substrates, thus 1 h was chosen as the standard for the following scope. It is worth mentioning that a lower excess of substrate at 20 minutes residence time gave much lower yields of **2** (63 and 28% for 5 and 1 equiv, respectively).

**Scheme 3:**
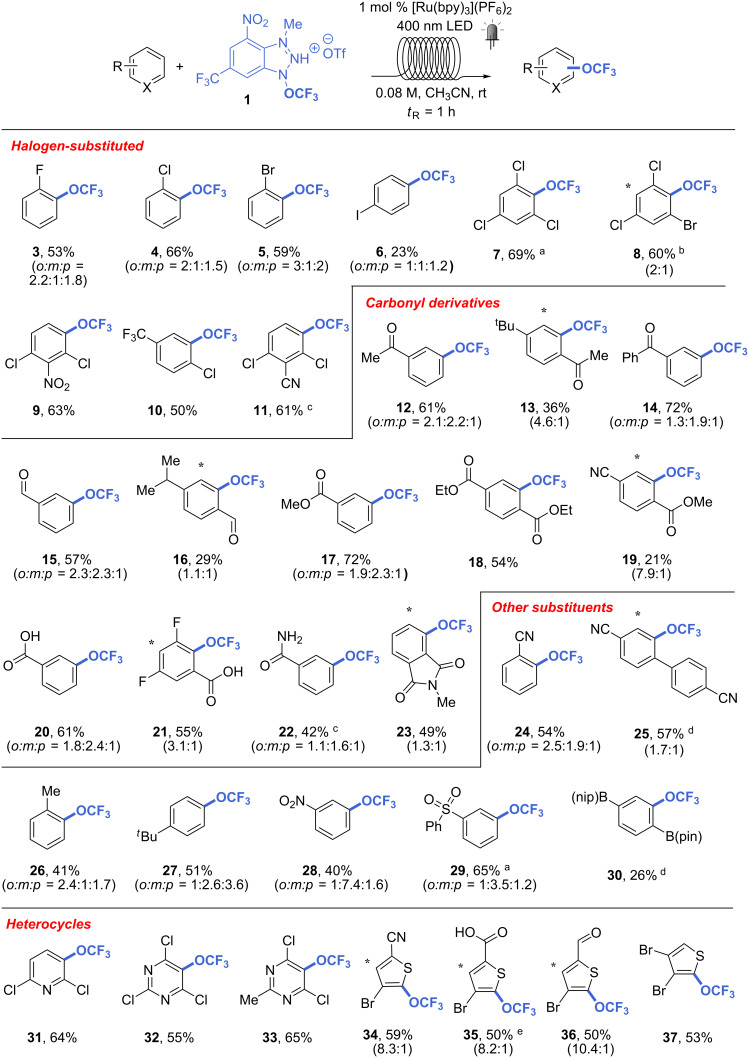
Scope of photoredox trifluoromethoxylation in continuous-flow. In case of different products, the major isomer is shown. The position of the CF_3_O group in the minor isomer is marked with a star. ^19^F NMR yields are reported. ^a^ CH_3_CN/CH_2_Cl_2_ 3:2, 0.08 M; ^b^ CH_3_CN/CH_2_Cl_2_ 1:1, 0.05 M; ^c^ CH_3_CN/CH_2_Cl_2_ 5:3, 0.05 M; ^d^ CH_3_CN/CH_2_Cl_2_ 3:2, 0.02 M; ^e^ CH_3_CN/CH_2_Cl_2_ 5:4, 0.057 M.

With the optimized conditions in hands, we proceeded to investigate the trifluoromethoxylation of different arenes and heteroarenes in acetonitrile. Solvent mixtures and lower concentrations had to be used in some cases due to solubility issues. As can be seen in Scheme 3, trifluoromethoxylation occurs preferentially at the most electron-rich site of the arenes, although sensitivity to steric hindrance was observed. The reactions with halogenated aryls occurred smoothly, leading to products **3**–**11** in good yields. An exception is represented by iodobenzene, which could be methoxylated in only 23% yield (product **6**). Similar results were obtained with carbonyl-containing compounds. Different aromatic ketones, aldehydes, esters, and carboxylic acids were transformed to the corresponding trifluoromethoxylated compounds (**12**–**23**) with generally good to high yields, except for **13**, **16**, **19** and **22** which were obtained in moderate or poor yields. Other synthetically useful substituents, including cyano, nitro, phenylsulphonyl, (pinacolato)boron, and alkyl groups could be tolerated in the reaction (**24**–**30**), leading to a yield range between 26 and 65%. The trifluoromethoxylation of pyridine, pyrimidine, and thiophene derivatives also took place smoothly, giving products **31**–**37** in good yields (50–65%).

Overall, the yields obtained for different products were similar to those reported by Ngai [24] and Togni [25] using batch protocols. In addition, in comparison with Ngai’s protocol, this flow method allows for a much reduced reaction (residence) time, therefore, demonstrating the potentials of this technology for the synthesis of trifluoromethoxylated compounds. The use of acetonitrile as solvent, rather than the CH_3_CN/CH_2_Cl_2_ mixture previously used [24], further increase the value of this protocol [32] for the synthesis of medicinally relevant molecules.

Based on the reaction mechanism proposed by Ngai and co-authors (Scheme 1C) [24], we hypothesized that the addition of base could potentially improve the reaction outcome. To prove this idea, we performed a series of experiments under batch and flow conditions (Table 2). Organic or soluble bases such as amines and tetrabutylammonium salts (Table 2, entries 1–5) proved unfortunately not successful, neither in batch nor flow, so we extended the investigation to inorganic bases. Among the bases tested, potassium hydrogenphosphates K_2_HPO_4_ and KH_2_PO_4_ led to increased yields, reaching up to 86% or 89% in batch (Table 2, entries 7 and 14). Because of the high yields obtained, the use of these bases in flow was investigated. The addition of water [33] allows these bases to be solubilized, preventing clogging; however, this strategy led to a mere 7% yield (Table 2, entry 8). During batch experiments we noted that after several minutes of irradiation, a homogeneous solution was obtained. Thus, we thought of using this trick for the flow process: irradiation of the reaction mixture in batch for 5 minutes, followed by injection into the microcapillary, provided **2** in 75% yield (Table 2, entry 15).

**Table 2:** Influence of bases on the photoredox trifluoromethoxylation in batch and flow.

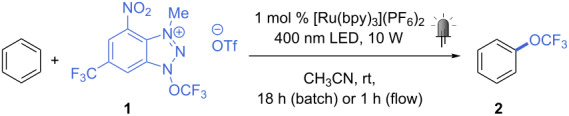				

Entry	Equiv of substrate	Сonc. of **1** (M)	Base	Yield (%)^a^

organic/soluble bases				

1	10	0.08	(Bu_4_N)H_2_PO_4_, 1 equiv	20
2	10	0.08	(Bu_4_N)H_2_PO_4_, 1 equiv	6 (flow)
3	25	0.04	DIPEA, 1 equiv	18
4	25	0.04	DBU, 1 equiv	14 (flow)
5	25	0.04	(Bu_4_N)OAc, 1 equiv	0 (flow)

inorganic bases				

6	25	0.04	K_2_CO_3_, 1 equiv	54
7	25	0.04	K_2_HPO_4_, 1 equiv	86
8	25	0.04	K_2_HPO_4_, 1 equiv	7^b^ (flow)
9	10	0.04	K_2_HPO_4_, 1 equiv	68
10	10	0.08	K_2_HPO_4_, 1 equiv	78
11	10	0.08	K_2_HPO_4_, 0.5 equiv	78
12	10	0.08	K_2_HPO_4_, 0.25 equiv	77
13	10	0.08	K_2_HPO_4_, 2 equiv	70
**14**	**10**	**0.08**	**KH_2_PO_4_, 1 equiv**	**89**
**15**	**10**	**0.08**	**KH_2_PO_4_, 1 equiv**	**75^c^ (flow)**
16	10	0.08	KH_2_PO_4_, 0.5 equiv	64
17	10	0.08	KH_2_PO_4_, 0.25 equiv	63
18	10	0.08	KH_2_PO_4_, 2 equiv	78
19	10	0.08	K_3_PO_4_, 1 equiv	54

^a 19^F NMR yields are reported; ^b^ 100 equiv of H_2_O added; ^c^ 5 minutes of irradiation in batch before flow reaction.

**Figure 1:**
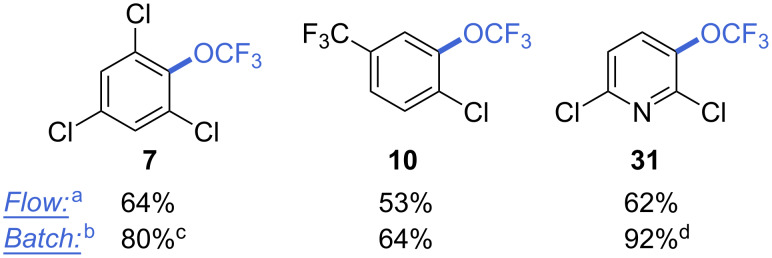
Effect of KH_2_PO_4_ – other substrates. ^a^ Conditions as for entry 15 (Table 2), 1 h residence time; ^b^ conditions as for entry 14 (Table 2), 18 h reaction time; ^c^ 0.5 equiv KH_2_PO_4_, CH_3_CN/CH_2_Cl_2_ 1:1; ^d^ 2 equiv KH_2_PO_4_. ^19^F NMR yields are reported.

These conditions were also applied to a few other substrates, for the sake of comparison (Figure 1).

Under flow conditions, products **7**, **10**, and **31** were obtained in yields comparable to the base-free method. However, the addition of KH_2_PO_4_ in batch results in considerably improved yields for the same compounds (64–92%). These experiments demonstrated that the addition of bases indeed represent an interesting modification of the previous protocol, despite being substrate-dependent.

## Conclusion

In summary, we have developed the first continuous-flow trifluoromethoxylation of arenes and heteroarenes. The use of flow photochemistry allows the reaction to occur 16 times faster than in batch, while at the same time achieving similar yields. Thanks to the intrinsic properties of microflow systems, especially for the scale-up of photochemical processes, we anticipate this procedure could be useful in medicinal chemistry applications. In addition, although no beneficial effect could be achieved in flow by the addition of bases, we proved this has a significant effect on the reaction in batch, leading to higher yields. This discovery will be useful for the development of future trifluoromethoxylation methodologies.

## Supporting Information

File 1 Procedure for continuous-flow and batch trifluoromethoxylation reactions and ^19^F NMR spectra of compounds **2**–**37**.
