# Supplementary material for: Photocatalytic trifluoromethoxylation of arenes and heteroarenes in continuous-flow
Source: Beilstein J Org Chem. 2020 Jun 15;16:1305–12. doi: 10.3762/bjoc.16.111 (PMC7308607; doi:10.3762/bjoc.16.111)
Supplement: File 1 — Procedure for continuous-flow and batch trifluoromethoxylation reactions and 19F NMR spectra of compounds 2–37. [file Beilstein_J_Org_Chem-16-1305-s001.pdf]

BEILSTEIN JOURNAL OF ORGANIC CHEMISTRY

Supporting Information

for

Photocatalytic trifluoromethoxylation of arenes and heteroarenes in continuous-flow

Alexander V. Nyuchev, Ting Wan, Borja Cendón, Carlo Sambiagio, Job J. C. Struijs, Michelle Ho, Moisés Gulías, Ying Wang and Timothy Noël

Beilstein J. Org. Chem. **2020,** *16,* 1305–1312. doi:10.3762/bjoc.16.111

Procedure for continuous-flow and batch trifluoromethoxylation reactions and ^{19}F NMR spectra of compounds 2–37

License and Terms: This is a supporting information file under the terms of the Creative Commons Attribution License (http://creativecommons.org/licenses/by/4.0). Please note that the reuse, redistribution and reproduction in particular requires that the authors and source are credited.
The license is subject to the *Beilstein Journal of Organic Chemistry* terms and conditions: (https://www.beilstein-journals.org/bjoc)

General information

All reagents and solvents were used as received without further purification. Reagents and solvents were bought from Sigma Aldrich, TCI, Fluorochem, ABCR, Acros and Alfa Aesar. Technical solvents were bought from VWR International and Biosolve, and are used as received. All capillary tubing and microfluidic fittings were purchased from IDEX Health & Science. Disposable syringes were from BD Discardit II® purchased from VWR Scientific. Syringe pumps were purchased from Chemix Inc., model Fusion 200 Touch. TLC analysis was performed using Silica on aluminum TLC plates (F254, Supelco Sigma-Aldrich™) with visualization under ultraviolet light (254 nm and 365 nm) or appropriate TLC staining. ^{19}F NMR (376 MHz) spectra were recorded at ambient temperature using a Bruker-Avance 400. Quantitative ^{19}F NMR spectra were recorded on 16 scans and d1 = 10 s, and reported in parts per million (ppm) downfield relative to internal standard $C_6H_5CF_3$ (63.30 ppm) [1]. NMR data was processed using the MestReNova 12.0.4-22023 software package. Known products were characterized by comparison with the corresponding ^{19}F-NMR from literature [1,2]. The ^{19}F NMR peak sometimes observed around -20 ppm corresponds to carbonyl difluoride (F_2CO) [3–5], generated by decomposition of the trifluoromethoxy radical [5]. The names of all products were generated using the PerkinElmer ChemBioDraw Ultra v.18.0.0.231 software package. LED strips were purchased from 123led B.V (The Netherlands); emission spectra shown in Figure S1.

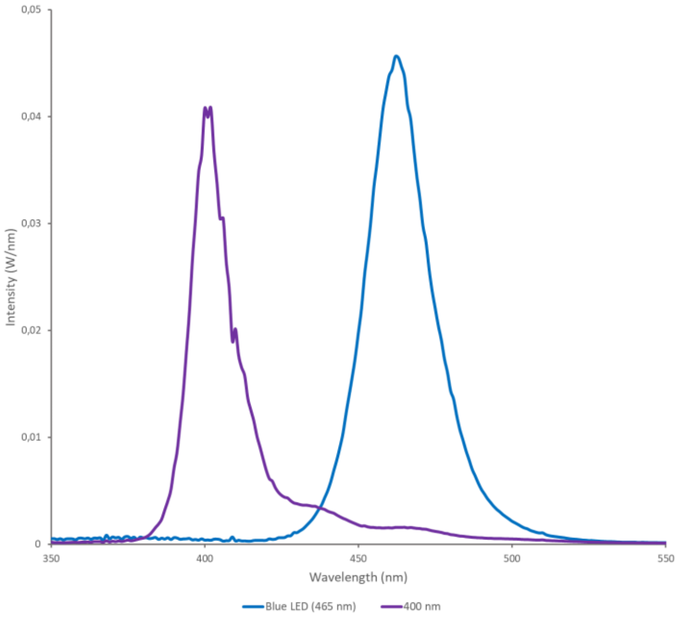

Figure S1. Emission spectra of the light sources.

Compound **1** (3-methyl-4-nitro-1-(trifluoromethoxy)-6-(trifluoromethyl)-1*H*-benzo[*d*][1,2,3]triazol-3-ium trifluoromethanesulfonate) was synthesized according published procedure [1]. Spectra were in full accordance with reported.

General procedure 1 (trifluoromethoxylation in continuous-flow)

The CF_3O-reagent **1** (1 equiv, 45.3 mg, 0.0944 mmol), $[Ru(bpy)_3](PF_6)_2$ (1 mol %, 0.9 mg) and solid substrate (10 equiv, 0.944 mmol) were added to a vial equipped with cap with septum and stirring bar. In case of liquid substrate, it was added after the solvent. Three vacuum-argon cycles were performed, and the vial was filled with argon. Dry acetonitrile (1.25 mL) was added via syringe. After 1 minute of vigorous sonification in ultrasonic bath, the solution was loaded into a 5 mL syringe. This syringe was then fitted to a syringe pump and connected to a 2.3 mL PFA microreactor coil (internal diameter of 750 µm), previously flushed with 5 mL of dry acetonitrile. The flowrate was set to 0.0383 mL/min to obtain a residence time of 1 h. When the syringe was fully empty, again dry acetonitrile was loaded into a syringe and injected to collect all product at the end of the reactor in a round bottom flask equipped with stirring bar and under argon atmosphere. 10 µL (11.9 mg, 0.0815 mmol) of benzotrifluoride ($C_6H_5CF_3$) were then added to the mixture and stirred for 1 minute. For quantitative ^{19}F NMR 0.3 mL of resulting solution was transferred to NMR tube and diluted with 0.3 mL of $CDCl_3$.

General procedure 2 (trifluoromethoxylation in batch conditions)

The CF_3O-reagent **1** (1 equiv, 48 mg, 0.1 mmol), $[Ru(bpy)_3](PF_6)_2$ (1 mol %, 0.9 mg), solid substrate (10 equiv, 1.0 mmol or 25 equiv, 2.5 mmol) and solid base (corresponding amount) were added to a 7 mL vial equipped with cap with septum and stirring bar. In case of liquid substrate and base, these were added after the solvent. Three vacuum-argon cycles were performed, and the vial was filled with argon. Dry acetonitrile (1.25 mL) was added via syringe. After 1 minute of vigorous sonification in ultrasonic bath, reaction was stirred and irradiated during 18 h. Then 10 µL (11.9 mg, 0.0815 mmol) of benzotrifluoride ($C_6H_5CF_3$) was added to the mixture and stirred for 1 minute. For quantitative ^{19}F NMR 0.3 mL of resulting solution was transferred to NMR tube and diluted with 0.3 mL of $CDCl_3$.

References

(1) Zheng, W.; Lee, J. W.; Morales-Rivera, C. A.; Liu, P.; Ngai, M. Y. *Angew. Chem., Int. Ed.* **2018**, *57*, 13795–13799. doi:10.1002/anie.201808495.

(2) Jelier, B. J.; Tripet, P. F.; Pietrasiak, E.; Franzoni, I.; Jeschke, G.; Togni, A. *Angew. Chem., Int. Ed.* **2018**, *57*, 13784–13789. doi:10.1002/anie.201806296.

(3) Lebedev, N. V.; Berenblit, V. V.; Troichanskaya, P. E.; Gubanov, V. A. *Russ. J. Appl. Chem.* **2009**, *82*, 449–455. doi:10.1134/S1070427209030197.

(4) Švec, P.; Eisner, A.; Kolářová, L.; Weidlich, T.; Pejchal, V.; Růžička, A. *Tetrahedron Lett.* **2008**, *49*, 6320–6323. doi:10.1016/j.tetlet.2008.08.060.

(5) Zheng, W.; Morales-Rivera, C. A.; Lee, J. W.; Liu, P.; Ngai, M. Y. *Angew. Chem., Int. Ed.* **2018**, *57*, 9645–9649. doi:10.1002/anie.201800598.

(Trifluoromethoxy)benzene (2).[1]

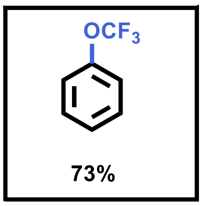

The reaction was carried out using benzene (73.7 mg, 0.944 mmol) as starting material according to the General procedure 1.

^{19}F NMR yield: 73%.

^{19}F NMR (376 MHz, $CDCl_3/CH_3CN$): δ (ppm) = -58.58 (s, 3F).

Compound is known [2].

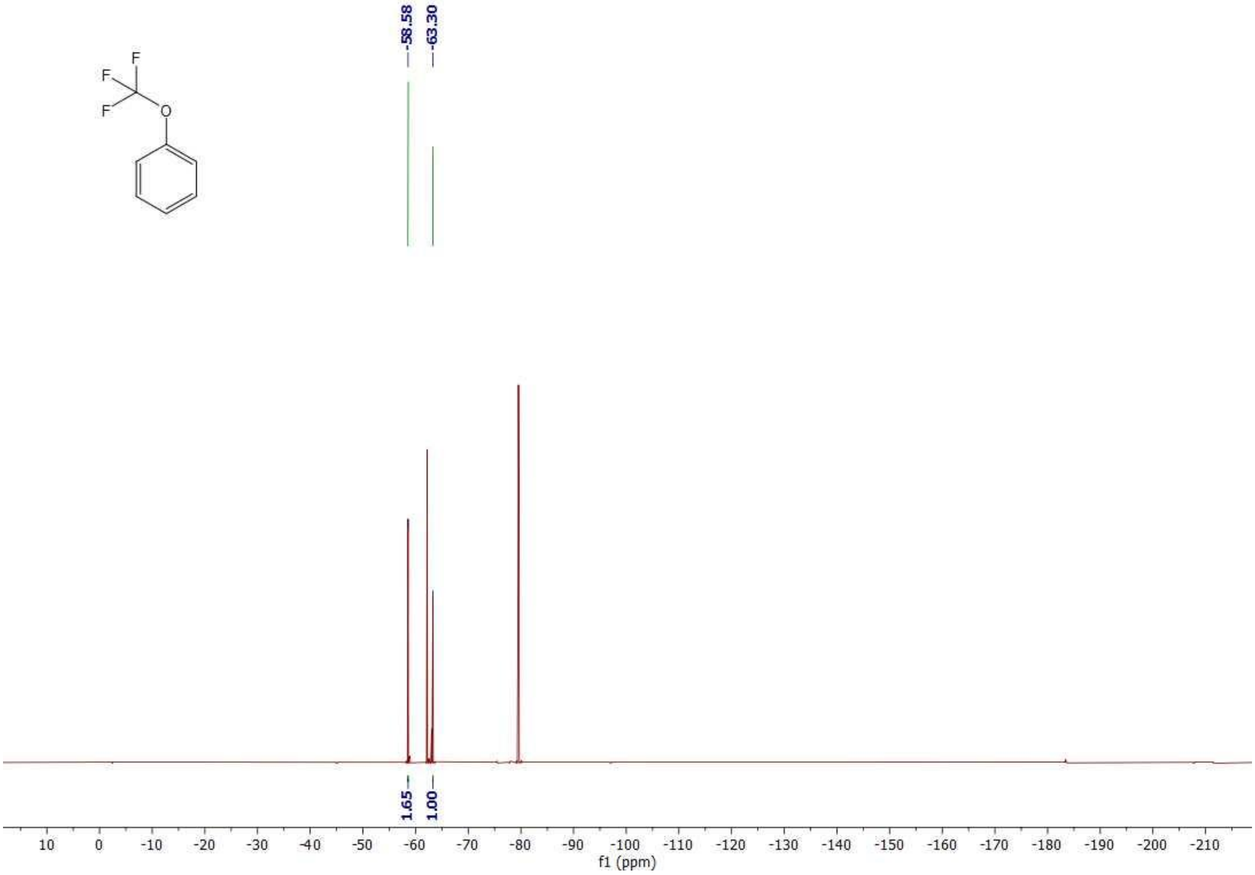

^{19}F NMR spectrum of **2**.

[1] In case of several products, name and structure (in frame) of the major isomer are given.

1-Fluoro-2-(trifluoromethoxy)benzene (3).

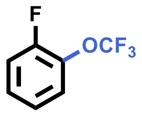

53%

(*o*:*m*:*p* = 2.2:1.0:1.8)

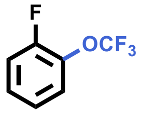

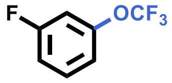

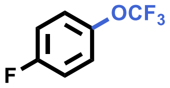

1-Fluoro-2-(trifluoromethoxy)benzene (*o*-isomer)	1-Fluoro-3-(trifluoromethoxy)benzene (*m*-isomer)	1-Fluoro-4-(trifluoromethoxy)benzene (*p*-isomer)

The reaction was carried out using fluorobenzene (90.7 mg, 0.944 mmol) as starting material according to the General procedure 1.

^{19}F NMR yield: 53% (*o* : *m* : *p* = 2.2 : 1.0 : 1.8).

^{19}F NMR *o*-isomer (376 MHz, $CDCl_3/CH_3CN$): δ (ppm) = -59.65 (d, *J* = 4.9 Hz, 3F), -130.90 – -131.12 (m, 1F).

^{19}F NMR *m*-isomer (376 MHz, $CDCl_3/CH_3CN$): δ (ppm) = -58.82 (s, 3F), -111.07 – -111.31 (m, 1F).

^{19}F NMR *p*-isomer (376 MHz, $CDCl_3/CH_3CN$): δ (ppm) = -59.16 (s, 3F), -116.14 – -116.21 (m, 1F).

Compounds are known [1,2].

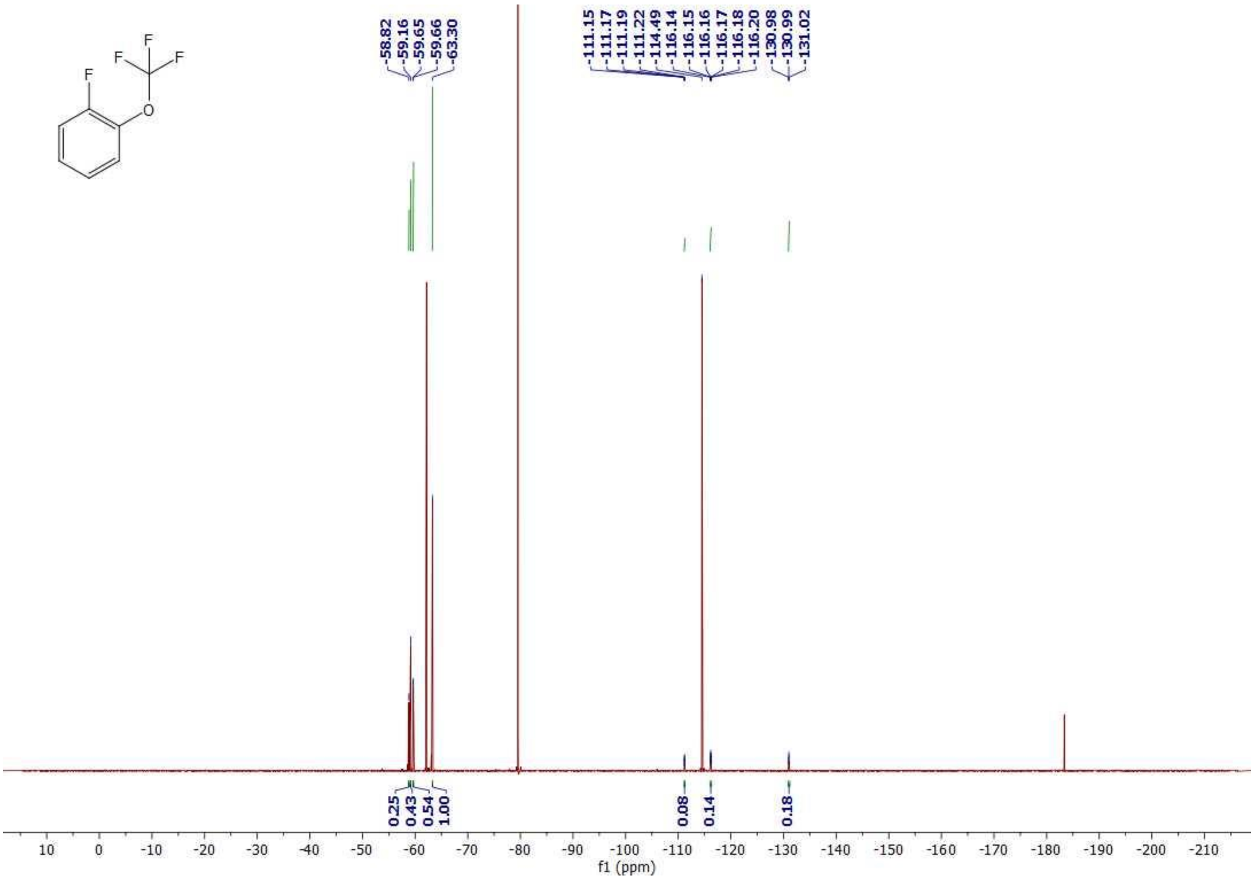

^{19}F NMR spectrum of **3**.

1-Chloro-2-(trifluoromethoxy)benzene (4).

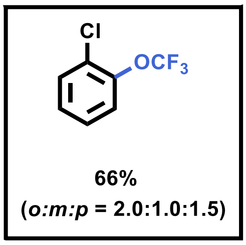

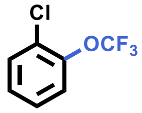

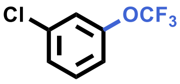

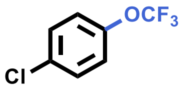

1-Chloro-2-(trifluoromethoxy)benzene (*o*-isomer) | **1-Chloro-3-(trifluoromethoxy)benzene (*m*-isomer)** | **1-Chloro-4-(trifluoromethoxy)benzene (*p*-isomer)**

The reaction was carried out using chlorobenzene (106.3 mg, 0.944 mmol) as starting material according to the General procedure 1.

^{19}F NMR yield: 66% (*o* : *m* : *p* = 2.0 : 1.0 : 1.5).

^{19}F NMR *o*-isomer (376 MHz, $CDCl_3/CH_3CN$): δ (ppm) = -58.64 (s, 3F).

^{19}F NMR *m*-isomer (376 MHz, $CDCl_3/CH_3CN$): δ (ppm) = -58.72 (s, 3F).

^{19}F NMR *p*-isomer (376 MHz, $CDCl_3/CH_3CN$): δ (ppm) = -58.89 (s, 3F).

Compounds are known [1,2].

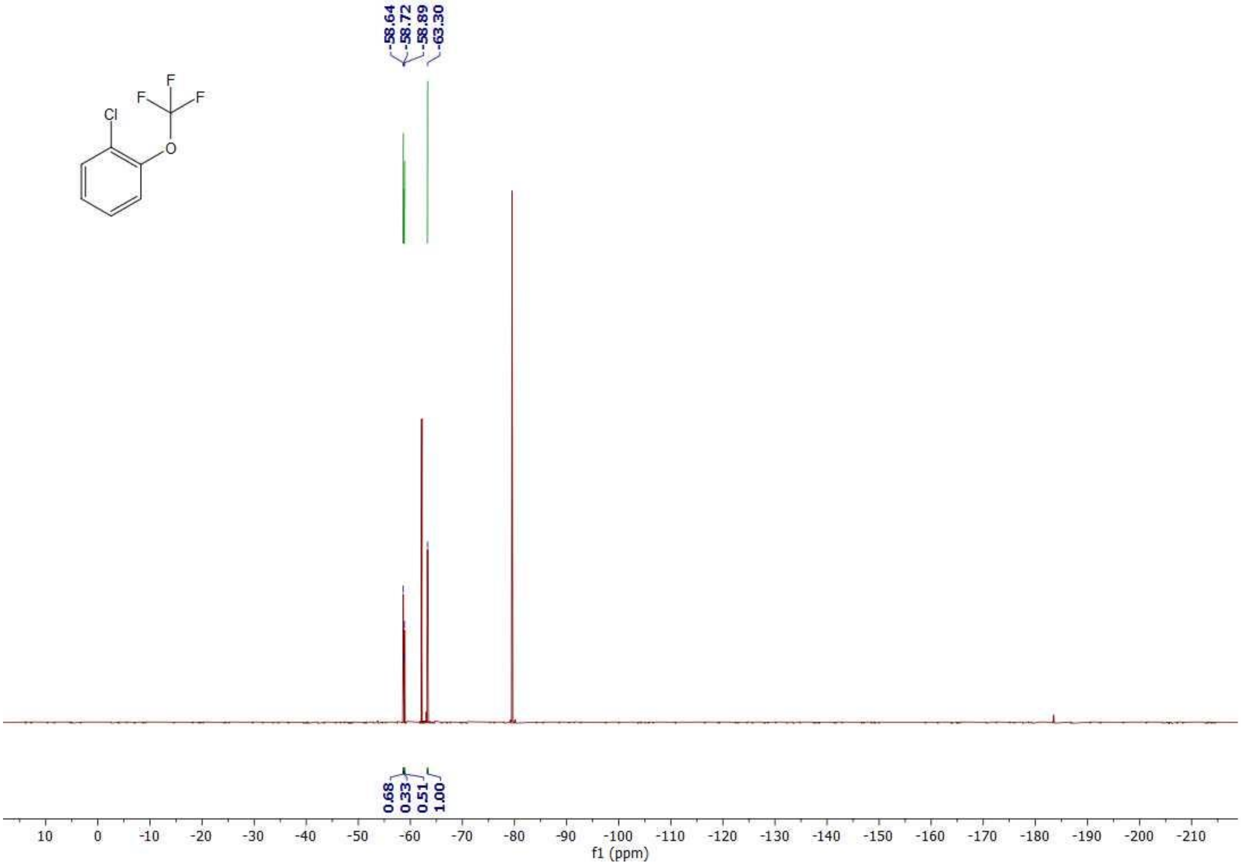

^{19}F NMR spectrum of **4**.

1-Bromo-2-(trifluoromethoxy)benzene (5).

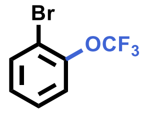

59%
(o:m:p = 3.0:1.0:2.0)

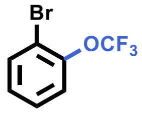

1-Bromo-2-(trifluoromethoxy)benzene (*o*-isomer)

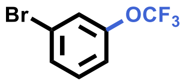

1-Bromo-3-(trifluoromethoxy)benzene (*m*-isomer)

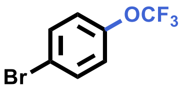

1-Bromo-4-(trifluoromethoxy)benzene (*p*-isomer)

The reaction was carried out using bromobenzene (147.6 mg, 0.944 mmol) as starting material according to the General procedure 1.

^{19}F NMR yield: 59% (o : m : p = 3.0 : 1.0 : 2.0).

^{19}F NMR *o*-isomer (376 MHz, $CDCl_3/CH_3CN$): δ (ppm) = -58.32 (s, 3F).

^{19}F NMR *m*-isomer (376 MHz, $CDCl_3/CH_3CN$): δ (ppm) =-58.72 (s, 3F).

^{19}F NMR *p*-isomer (376 MHz, $CDCl_3/CH_3CN$): δ (ppm) =-58.86 (s, 3F).

Compounds are known [1,2].

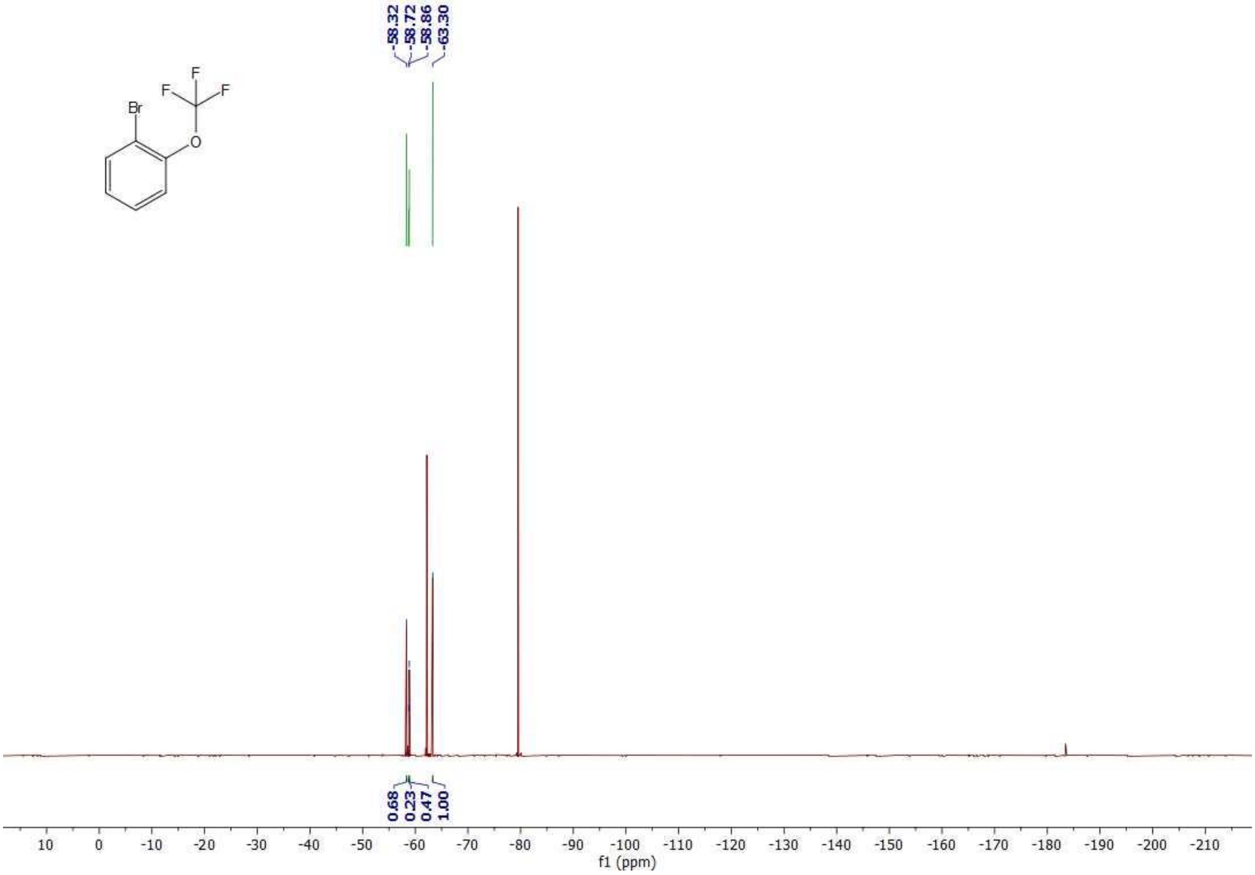

^{19}F NMR spectrum of **5**.

1-Iodo-4-(trifluoromethoxy)benzene (6).

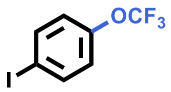

23%
(*o*:*m*:*p* = 1.0:1.0:1.2)

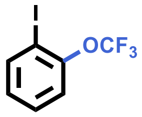

1-Iodo-2-(trifluoromethoxy)benzene (*o*-isomer)

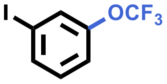

1-Iodo-3-(trifluoromethoxy)benzene (*m*-isomer)

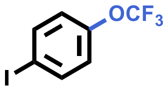

1-Iodo-4-(trifluoromethoxy)benzene (*p*-isomer)

The reaction was carried out using iodobenzene (192.6 mg, 0.944 mmol) as starting material according to the General procedure 1.

^{19}F NMR yield: 23% (*o* : *m* : *p* = 1.0 : 1.0 : 1.2).

^{19}F NMR *o*-isomer (376 MHz, $CDCl_3/CH_3CN$): δ (ppm) = -57.79 (s, 3F).

^{19}F NMR *m*-isomer (376 MHz, $CDCl_3/CH_3CN$): δ (ppm) = -58.70 (s, 3F).

^{19}F NMR *p*-isomer (376 MHz, $CDCl_3/CH_3CN$): δ (ppm) = -58.81 (s, 3F).

Compound is known [2].

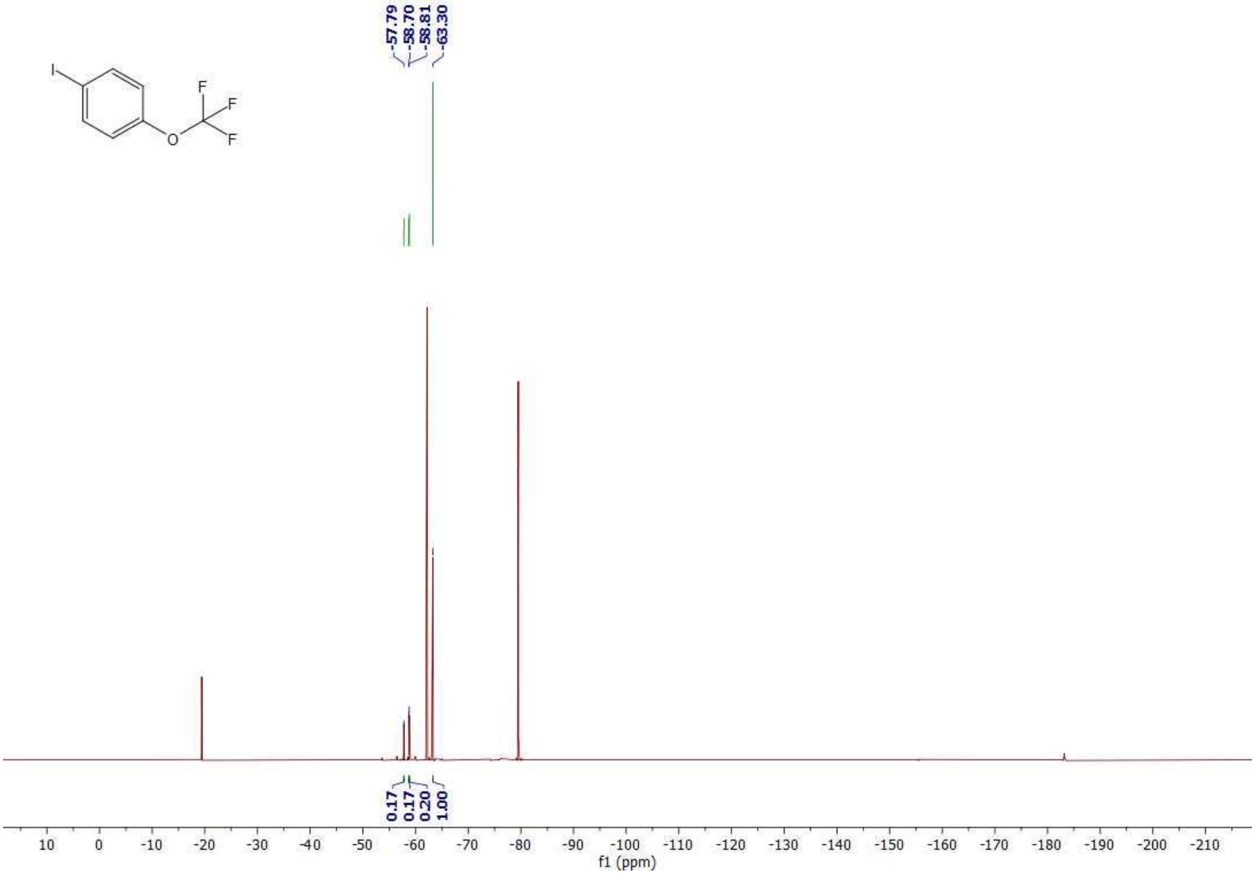

^{19}F NMR spectrum of **6**.

1,3,5-Trichloro-2-(trifluoromethoxy)benzene (7).

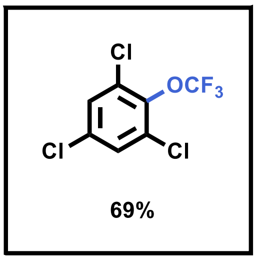

The reaction was carried out using 1,3,5-trichlorobenzene (171.3 mg, 0.944 mmol) as starting material according to the General procedure 1.

^{19}F NMR yield: 69%.

^{19}F NMR (376 MHz, $CDCl_3/CH_3CN$): δ (ppm) = -56.95 (s, 3F).

Compound is known [1].

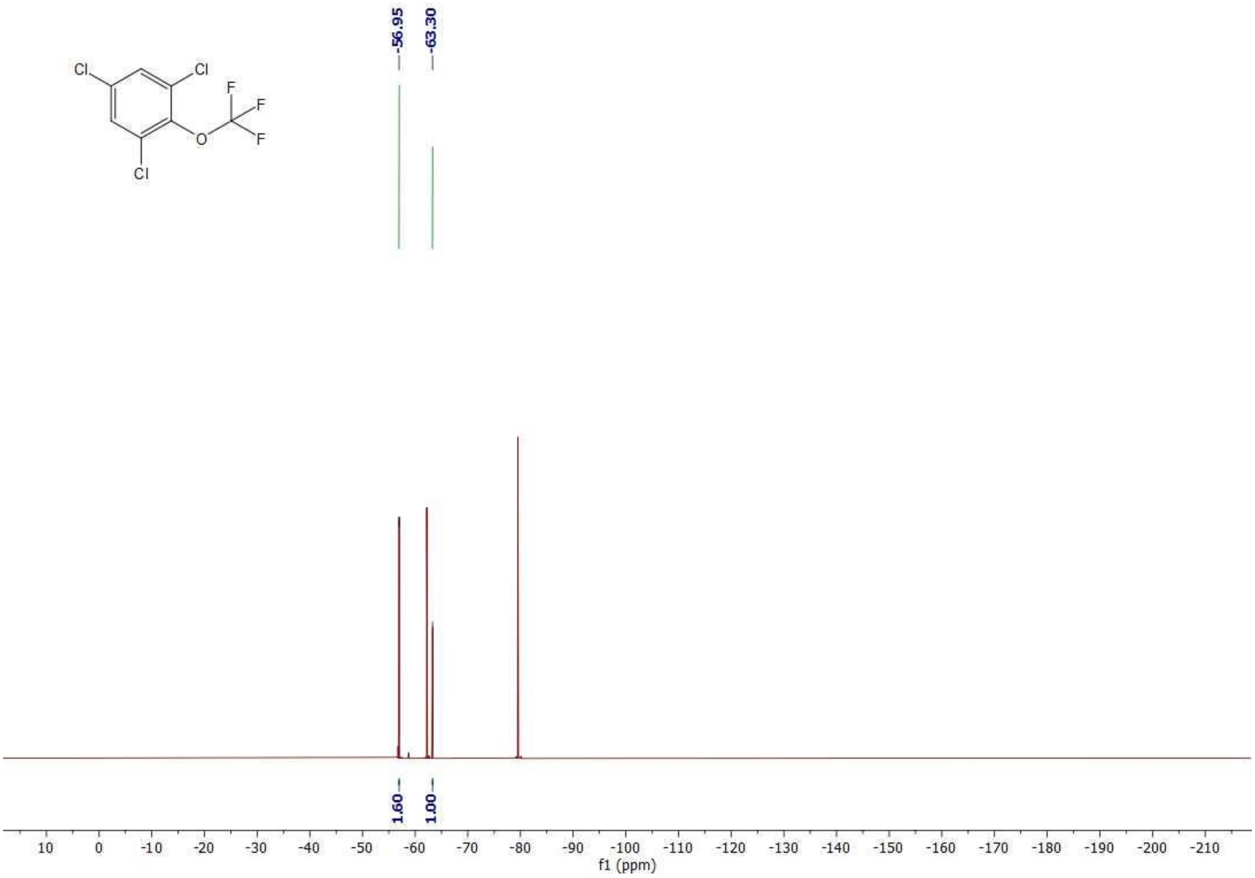

^{19}F NMR spectrum of **7**.

1-Bromo-3,5-dichloro-2-(trifluoromethoxy)benzene (8).

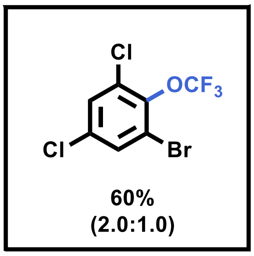

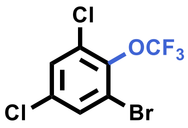

1-Bromo-3,5-dichloro-2-(trifluoromethoxy)benzene
major

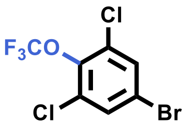

5-Bromo-1,3-dichloro-2-(trifluoromethoxy)benzene
minor

The reaction was carried out using 1-bromo-3,5-dichlorobenzene (213.2 mg, 0.944 mmol) as starting material according to the General procedure 1.

^{19}F NMR yield: 60% (2.0 : 1.0).

^{19}F NMR major isomer (376 MHz, $CDCl_3/CH_3CN$): δ (ppm) = -56.27 (s, 3F,).

^{19}F NMR major isomer (376 MHz, $CDCl_3/CH_3CN$): δ (ppm) = -56.93 (s, 3F).

Compound is known [1].

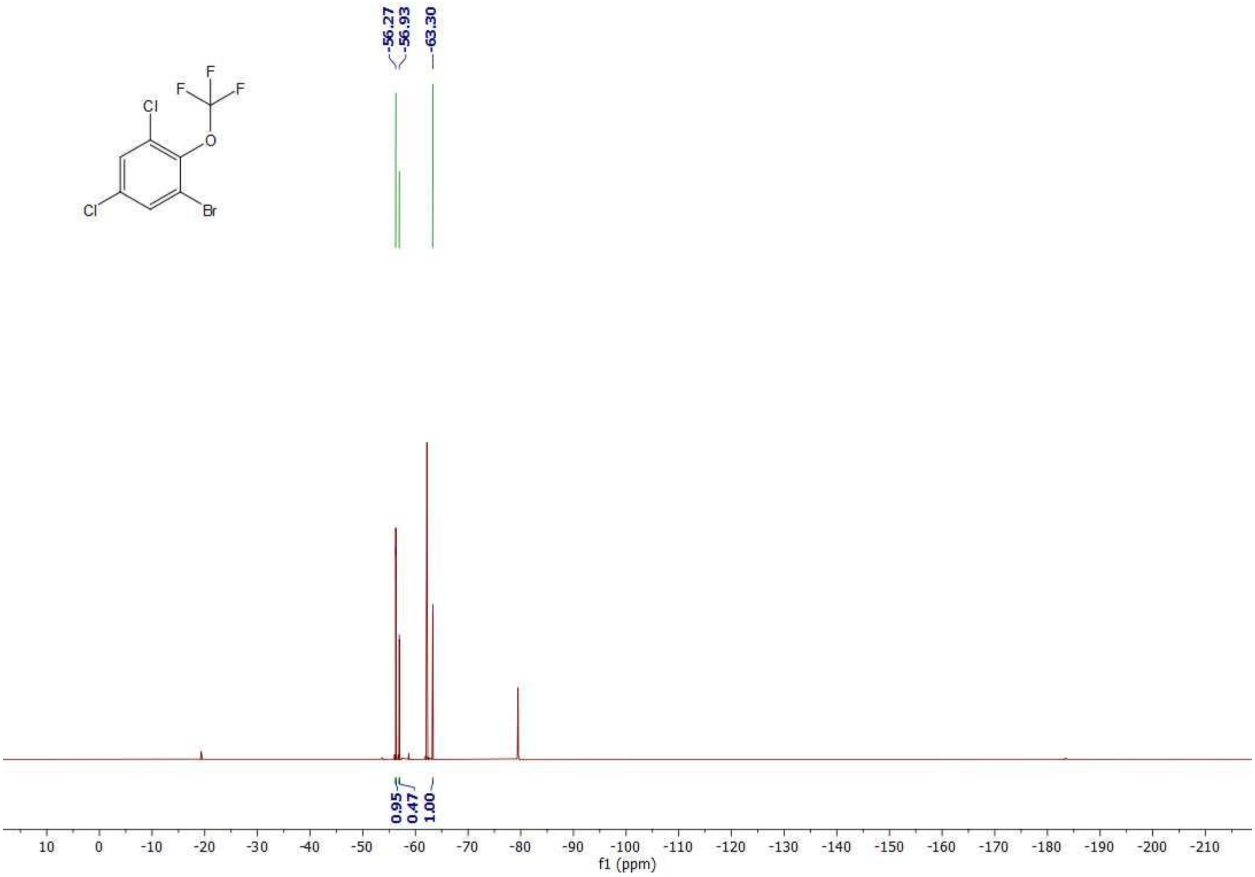

^{19}F NMR spectrum of **8**.

1,3-Dichloro-2-nitro-4-(trifluoromethoxy)benzene (9).

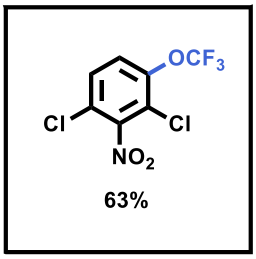

The reaction was carried out using 1,3-dichloro-2-nitrobenzene (181.2 mg, 0.944 mmol) as starting material according to the General procedure 1.

^{19}F NMR yield: 63%.

^{19}F NMR (376 MHz, $CDCl_3/CH_3CN$): δ (ppm) = -58.82 (s, 3F).

Compound is known [2].

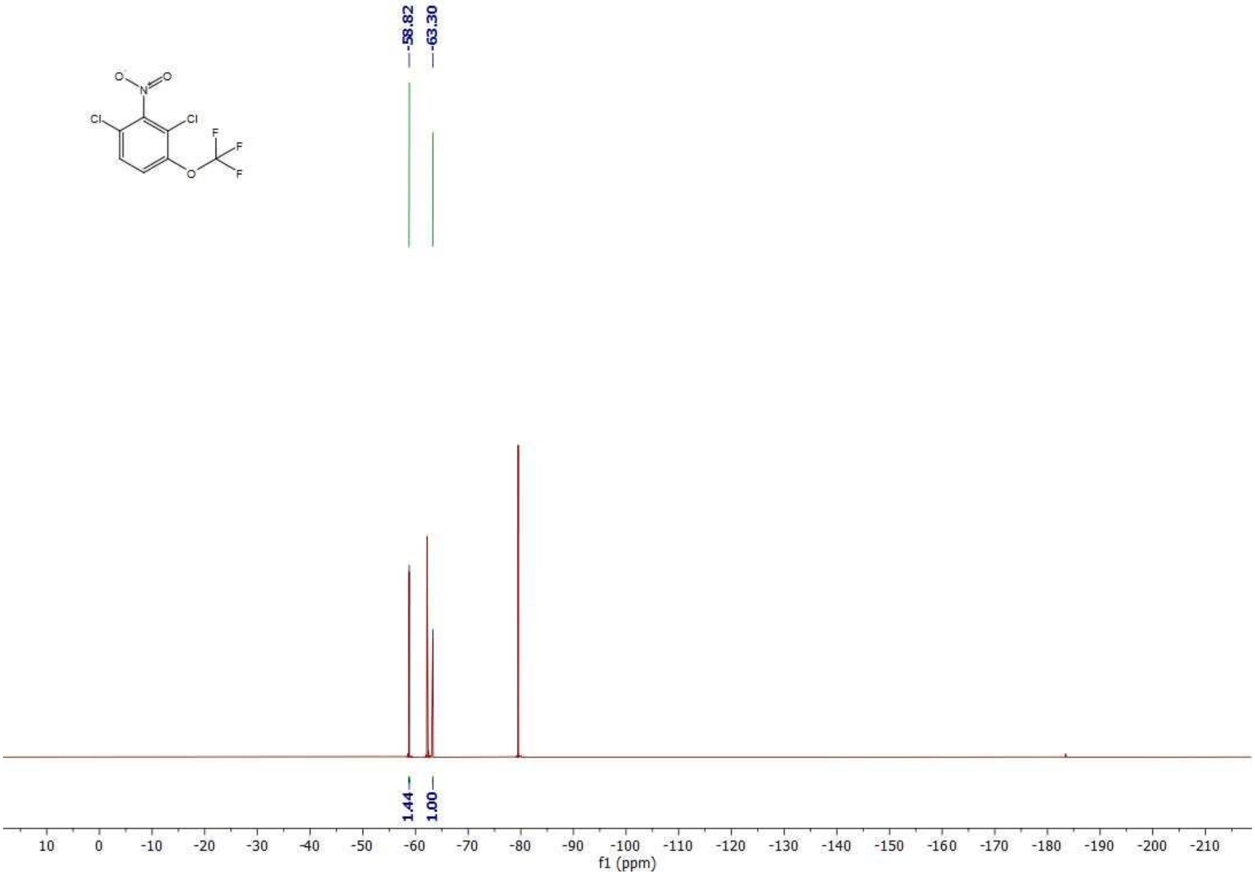

^{19}F NMR spectrum of **9**.

1-Chloro-2-(trifluoromethoxy)-4-(trifluoromethyl)benzene (10).

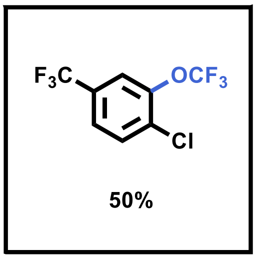

The reaction was carried out using 1-chloro-4-(trifluoromethyl)benzene (170.5 mg, 0.944 mmol) as starting material according to the General procedure 1.

^{19}F NMR yield: 50%.

^{19}F NMR (376 MHz, $CDCl_3/CH_3CN$): δ (ppm) = -58.79 (s, 3F), -63.39 (s, 3F).

Compound is known [1].

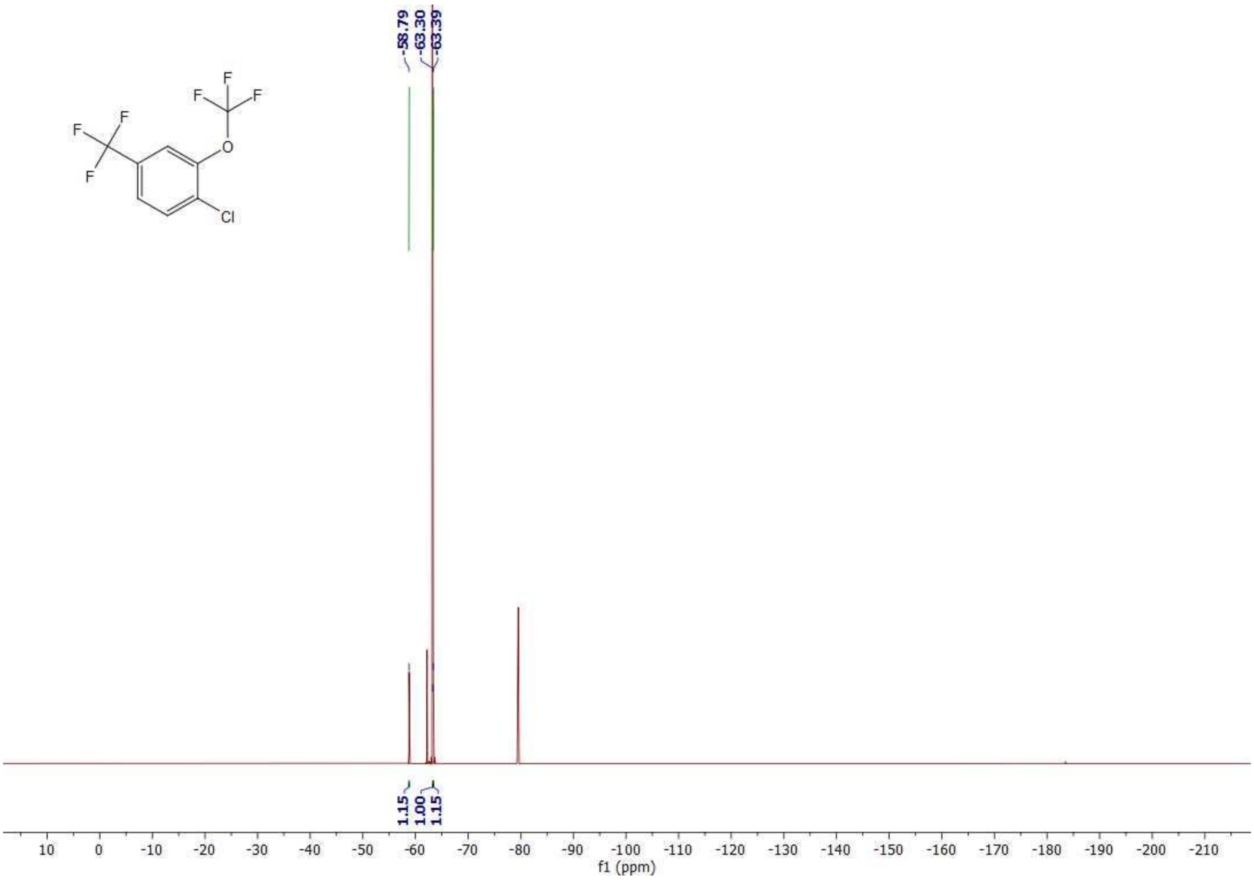

^{19}F NMR spectrum of **10**.

2,6-Dichloro-3-(trifluoromethoxy)benzonitrile (11).

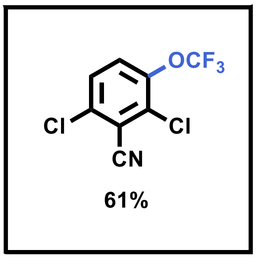

The reaction was carried out using 2,6-dichlorobenzonitrile (162.4 mg, 0.944 mmol) as starting material according to the General procedure 1.

^{19}F NMR yield: 61%.

^{19}F NMR (376 MHz, $CDCl_3/CH_3CN$): δ (ppm) = -58.75 (s, 3F).

Compound is known [2].

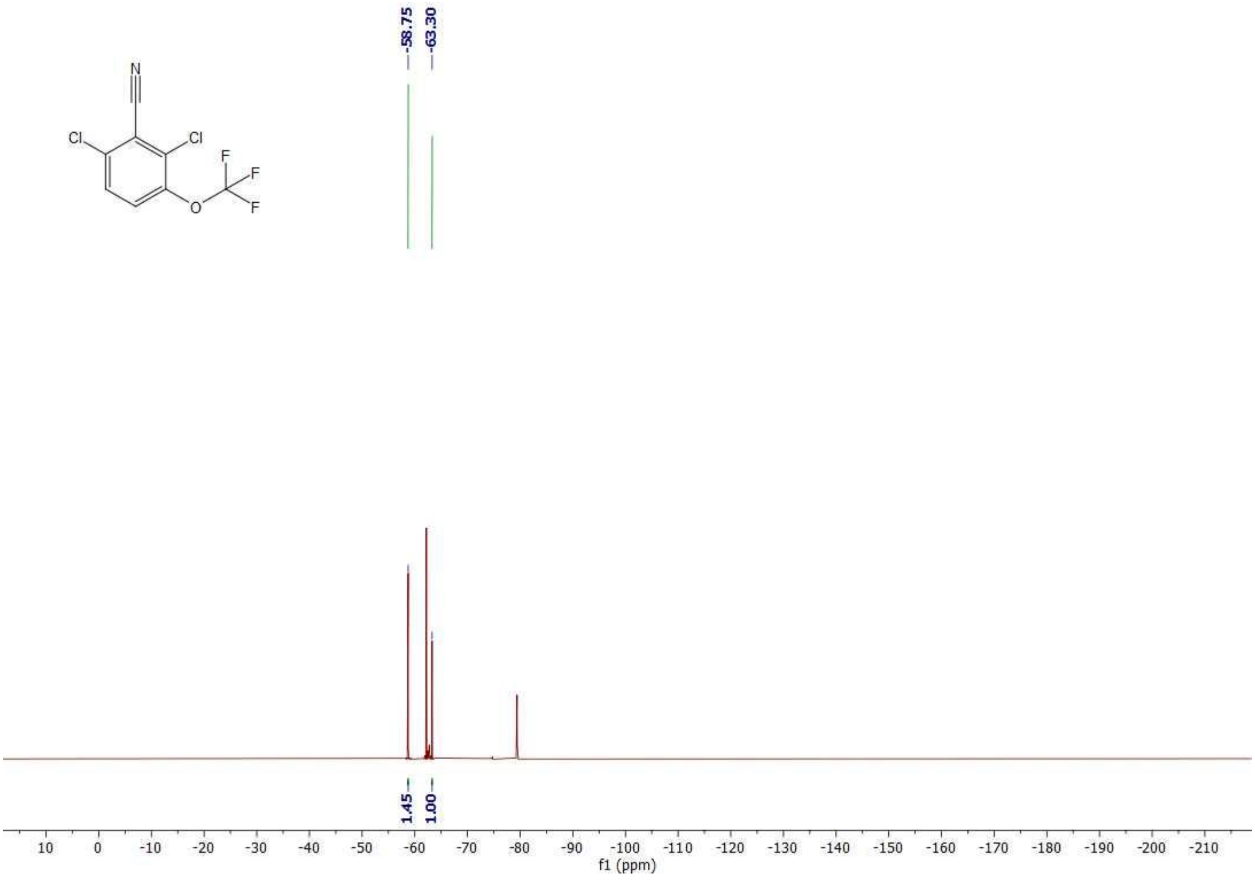

^{19}F NMR spectrum of **11**.

1-(3-(Trifluoromethoxy)phenyl)ethan-1-one (12).

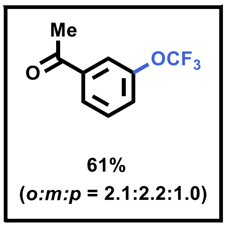

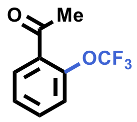

1-(2-(Trifluoromethoxy)phenyl)ethan-1-one (*o*-isomer)

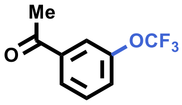

1-(3-(Trifluoromethoxy)phenyl)ethan-1-one (*m*-isomer)

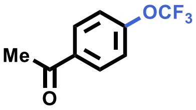

1-(4-(Trifluoromethoxy)phenyl)ethan-1-one (*p*-isomer)

The reaction was carried out using acetophenone (113.5 mg, 0.944 mmol) as starting material according to the General procedure 1.

^{19}F NMR yield: 61% (*o* : *m* : *p* = 2.1 : 2.2 : 1.0).

^{19}F NMR *o*-isomer (376 MHz, $CDCl_3/CH_3CN$): δ (ppm) = -57.83 (s, 3F).

^{19}F NMR *m*-isomer (376 MHz, $CDCl_3/CH_3CN$): δ (ppm) = -58.62 (s, 3F).

^{19}F NMR *p*-isomer (376 MHz, $CDCl_3/CH_3CN$): δ (ppm) = -58.35 (s, 3F).

Compounds are known [1,2].

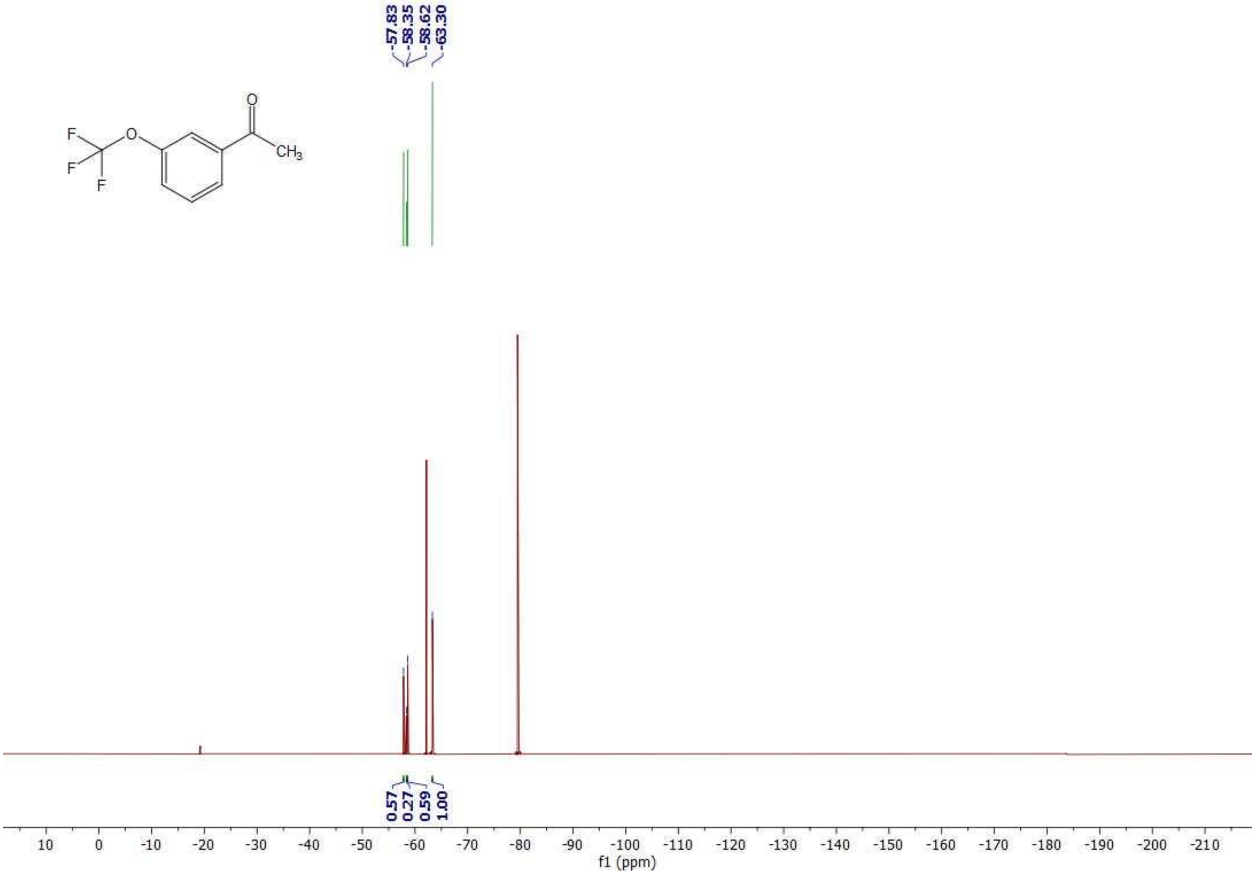

^{19}F NMR spectrum of **12**.

1-(4-(*tert*-Butyl)-2-(trifluoromethoxy)phenyl)ethan-1-one (13).

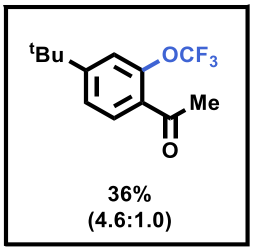

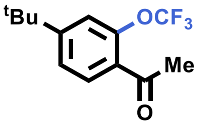

1-(4-(*tert*-Butyl)-2-(trifluoromethoxy)phenyl)ethan-1-one
major

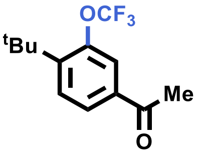

1-(4-(*tert*-Butyl)-3-(trifluoromethoxy)phenyl)ethan-1-one
minor

The reaction was carried out using 1-(4-(*tert*-butyl)phenyl)ethan-1-one (166.4 mg, 0.944 mmol) as starting material according to the General procedure 1.

^{19}F NMR yield: 36% (4.6 : 1.0).

^{19}F NMR major isomer (376 MHz, $CDCl_3/CH_3CN$): δ (ppm) = -57.74 (d, *J* = 1.4 Hz, 3F).

^{19}F NMR minor isomer (376 MHz, $CDCl_3/CH_3CN$): δ (ppm) =-55.37 (d, *J* = 1.9 Hz, 3F).

Compounds are known [2].

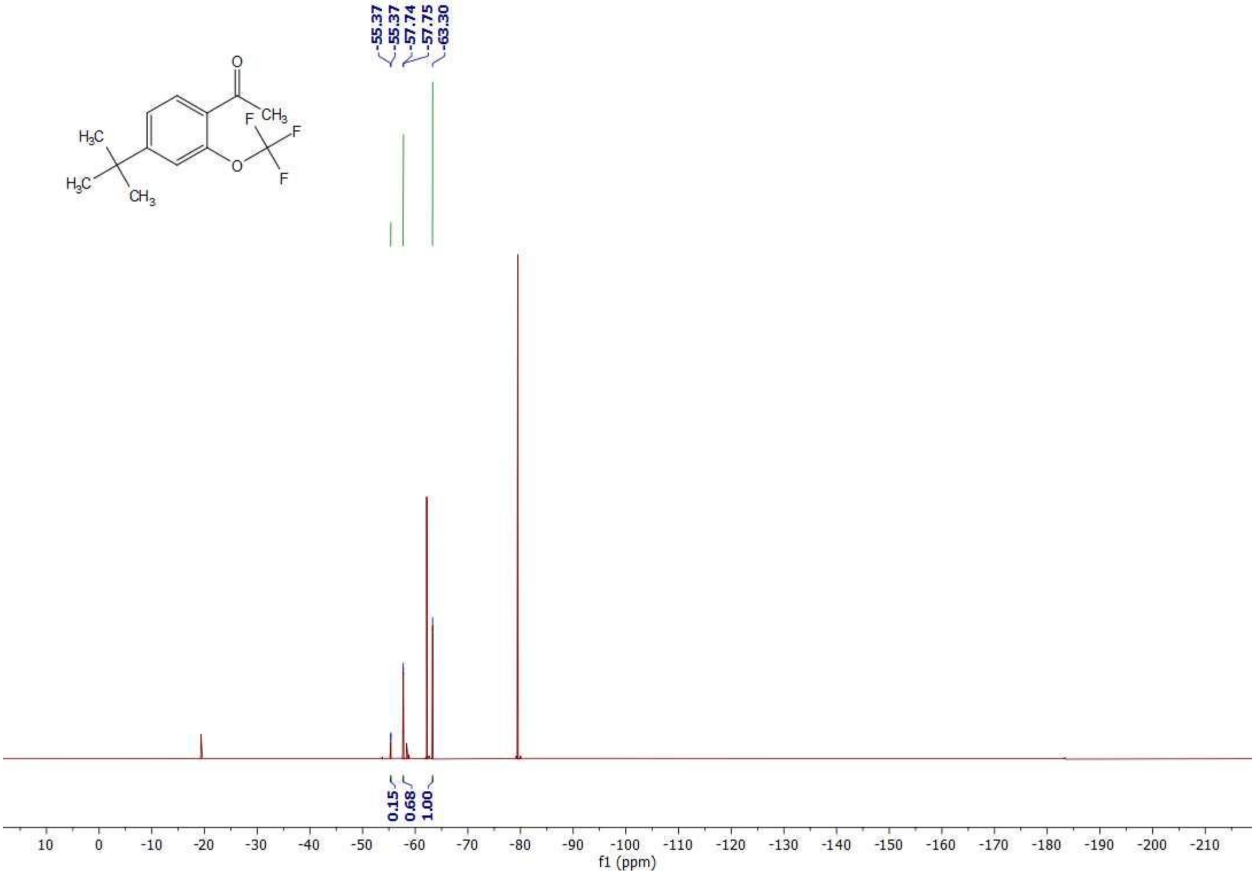

^{19}F NMR spectrum of **13**.

Phenyl(3-(trifluoromethoxy)phenyl)methanone (14).

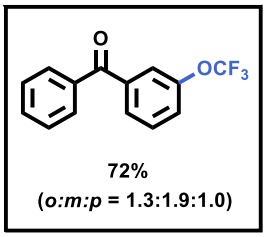

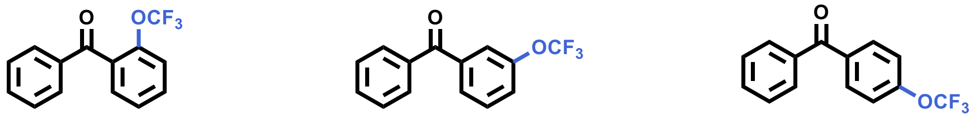

Phenyl(2-(trifluoromethoxy)phenyl) methanone (*o*-isomer) | **Phenyl(3-(trifluoromethoxy)phenyl) methanone (*m*-isomer)** | **Phenyl(4-(trifluoromethoxy)phenyl) methanone (*p*-isomer)**

The reaction was carried out using benzophenone (172.0 mg, 0.944 mmol) as starting material according to the General procedure 1.

^{19}F NMR yield: 72% (*o* : *m* : *p* = 1.3 : 1.9 : 1.0).

^{19}F NMR *o*-isomer (376 MHz, $CDCl_3/CH_3CN$): δ (ppm) = -58.16 (d, *J* = 2.1 Hz, 3F).

^{19}F NMR *m*-isomer (376 MHz, $CDCl_3/CH_3CN$): δ (ppm) = -58.61 (s, 3F).

^{19}F NMR *p*-isomer (376 MHz, $CDCl_3/CH_3CN$): δ (ppm) =-58.34 (s, 3F).

Compounds are known [2].

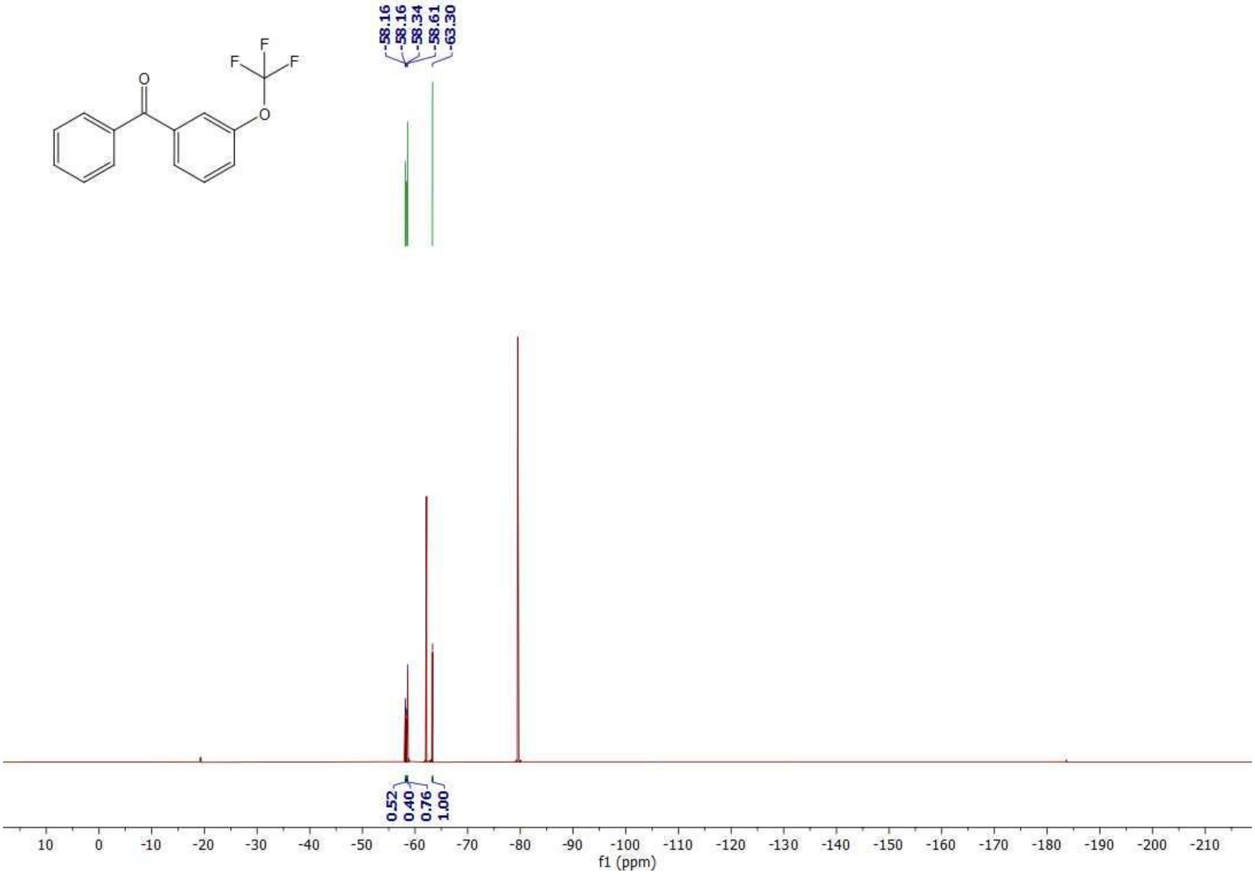

^{19}F NMR spectrum of **14**.

3-(Trifluoromethoxy)benzaldehyde (15).

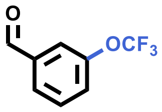

57%
(*o*:*m*:*p* = 2.3:2.3:1.0)

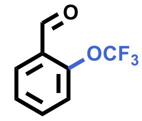

2-(Trifluoromethoxy)benzaldehyde (*o*-isomer)

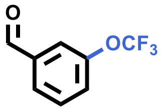

3-(Trifluoromethoxy)benzaldehyde (*m*-isomer)

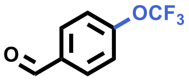

4-(Trifluoromethoxy)benzaldehyde (*p*-isomer)

The reaction was carried out using benzaldehyde (100.2 mg, 0.944 mmol) as starting material according to the General procedure 1.

^{19}F NMR yield: 57% (*o* : *m* : *p* = 2.3 : 2.3 : 1.0).

^{19}F NMR *o*-isomer (376 MHz, $CDCl_3/CH_3CN$): δ (ppm) = -58.47 (d, *J* = 1.7 Hz, 3F).

^{19}F NMR *m*-isomer (376 MHz, $CDCl_3/CH_3CN$): δ (ppm) = -58.71 (s, 3F).

^{19}F NMR *p*-isomer (376 MHz, $CDCl_3/CH_3CN$): δ (ppm) =-58.36 (s, 3F).

Compounds are known [2].

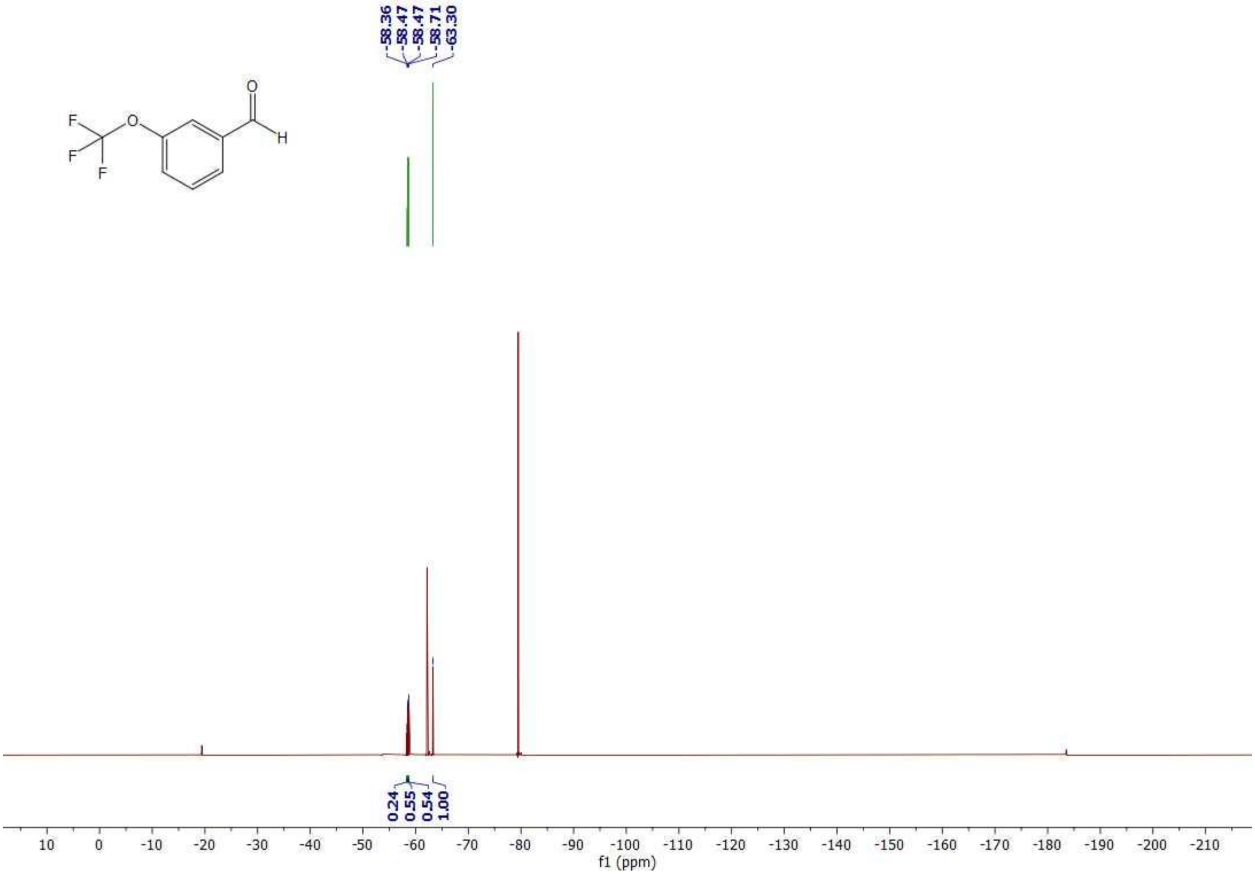

^{19}F NMR spectrum of **15**.

4-Isopropyl-2-(trifluoromethoxy)benzaldehyde (16).

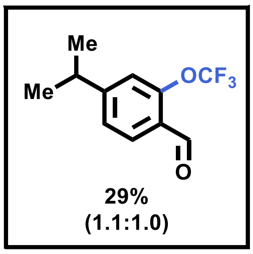

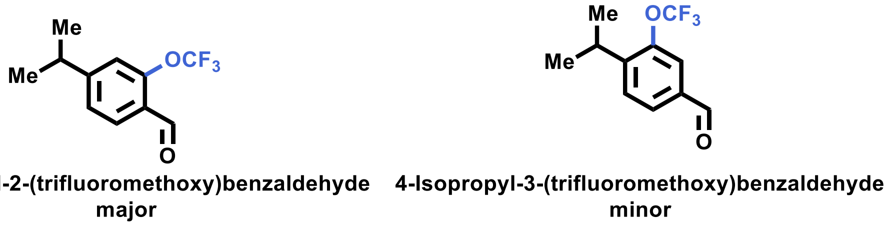

The reaction was carried out using 4-isopropylbenzaldehyde (139.9 mg, 0.944 mmol) as starting material according to the General procedure 1.

^{19}F NMR yield: 29% (1.1 : 1.0).

^{19}F NMR major isomer (376 MHz, $CDCl_3/CH_3CN$): δ (ppm) = -58.35 (d, J = 1.5 Hz, 3F).

^{19}F NMR minor isomer (376 MHz, $CDCl_3/CH_3CN$): δ (ppm) = -57,86 (d, J = 1.7 Hz, 3F).

Compounds are known [2].

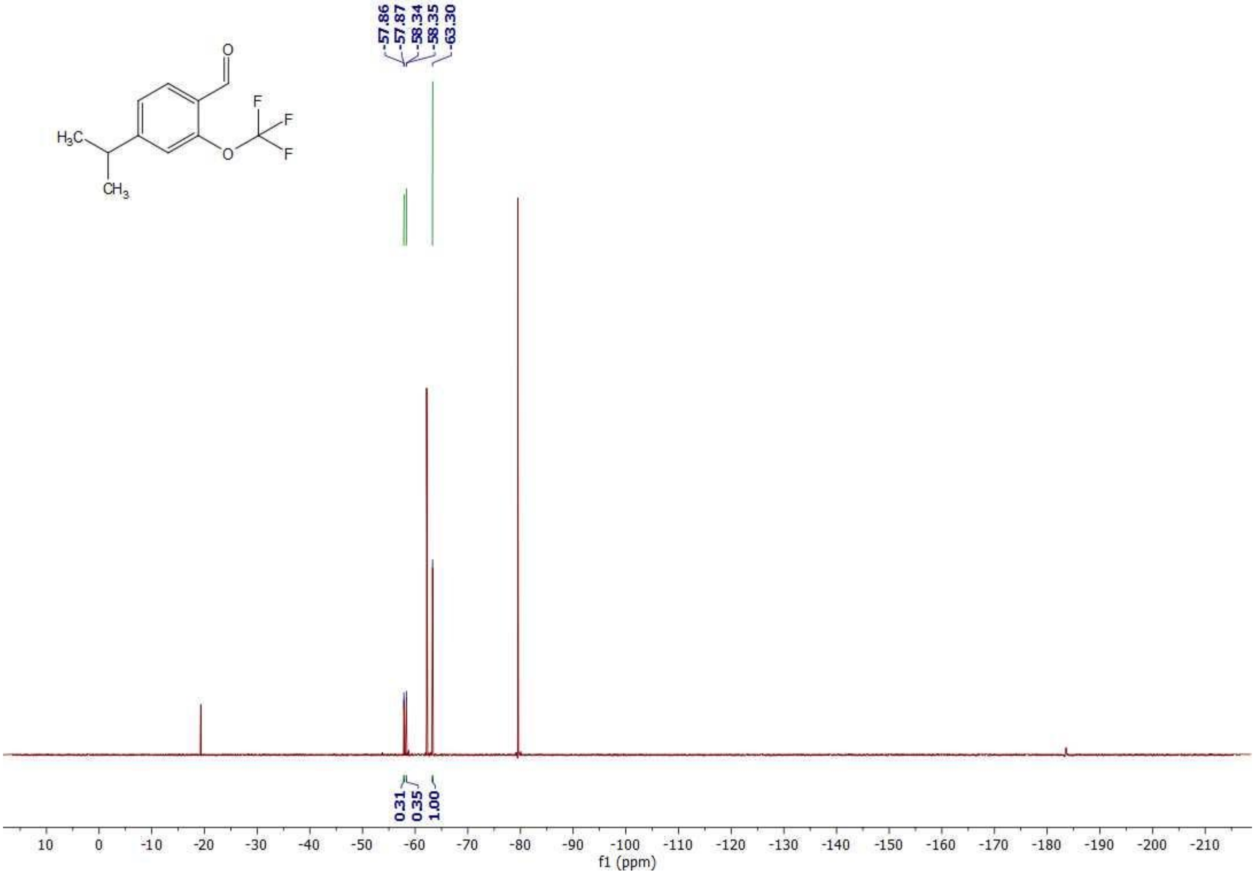

^{19}F NMR spectrum of **16**.

Methyl 3-(trifluoromethoxy)benzoate (17).

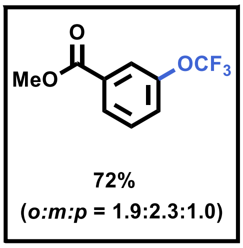

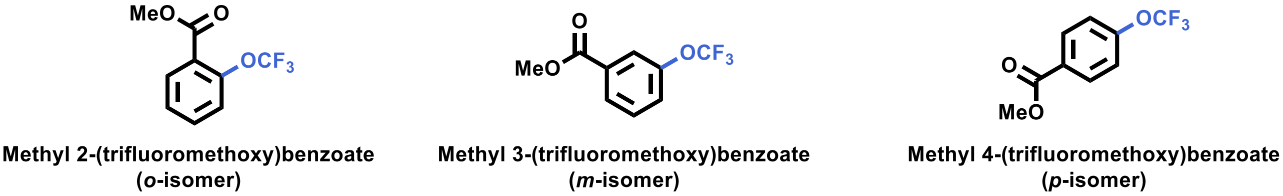

The reaction was carried out using methyl benzoate (128.6 mg, 0.944 mmol) as starting material according to the General procedure 1.

^{19}F NMR yield: 72% (*o* : *m* : *p* = 1.9 : 2.3 : 1.0).

^{19}F NMR *o*-isomer (376 MHz, $CDCl_3/CH_3CN$): δ (ppm) = -58.22 (s, 3F).

^{19}F NMR *m*-isomer (376 MHz, $CDCl_3/CH_3CN$): δ (ppm) = -58.68 (s, 3F).

^{19}F NMR *p*-isomer (376 MHz, $CDCl_3/CH_3CN$): δ (ppm) = -58.40 (s, 3F).

Compounds are known [1,2].

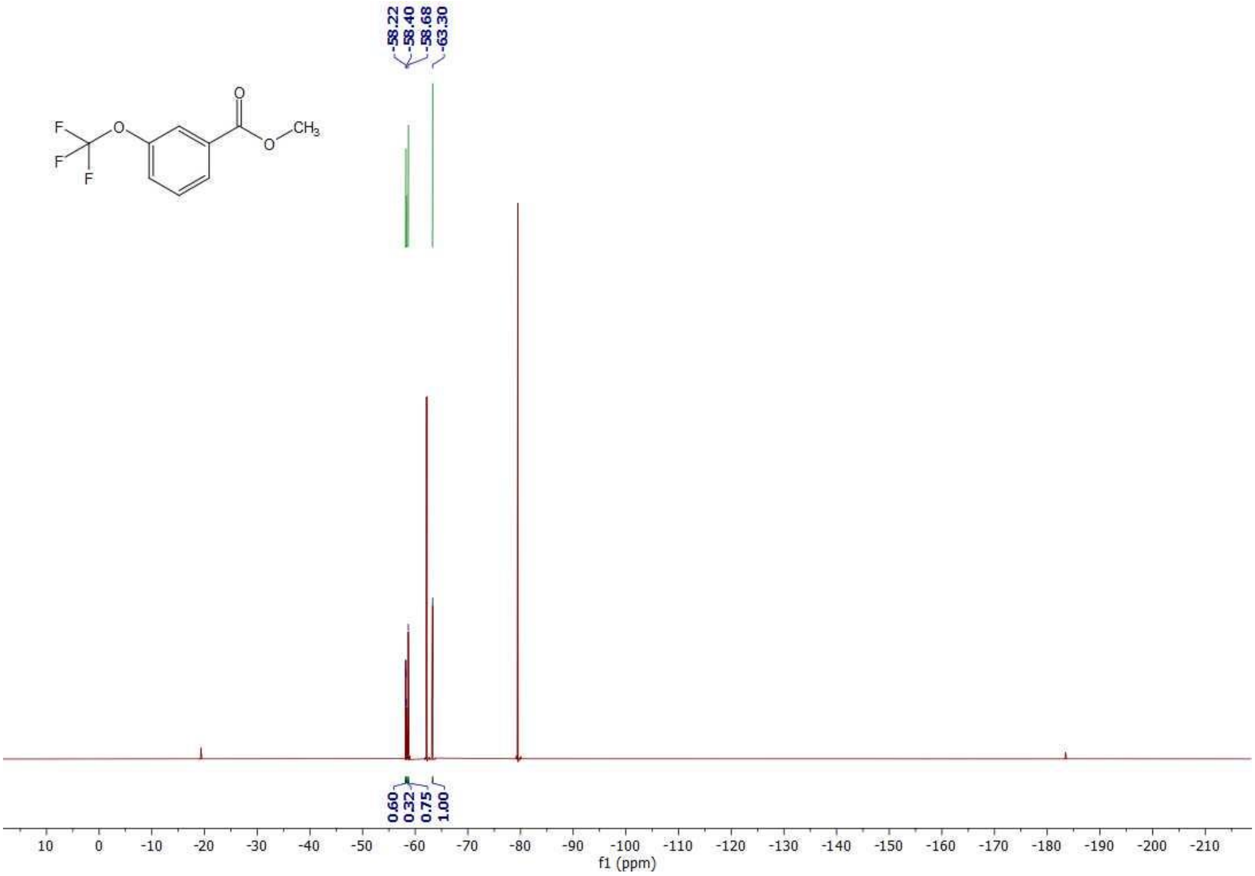

^{19}F NMR spectrum of **17**.

Diethyl 2-(trifluoromethoxy)terephthalate (18).

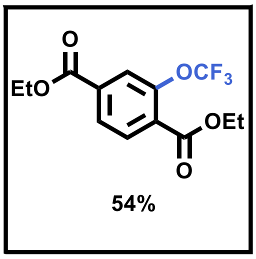

The reaction was carried out using diethyl terephthalate (208.9 mg, 0.944 mmol) as starting material according to the General procedure 1.

^{19}F NMR yield: 54%.

^{19}F NMR (376 MHz, $CDCl_3/CH_3CN$): δ (ppm) = -58.21 (s, 3F).

Compound is known [2].

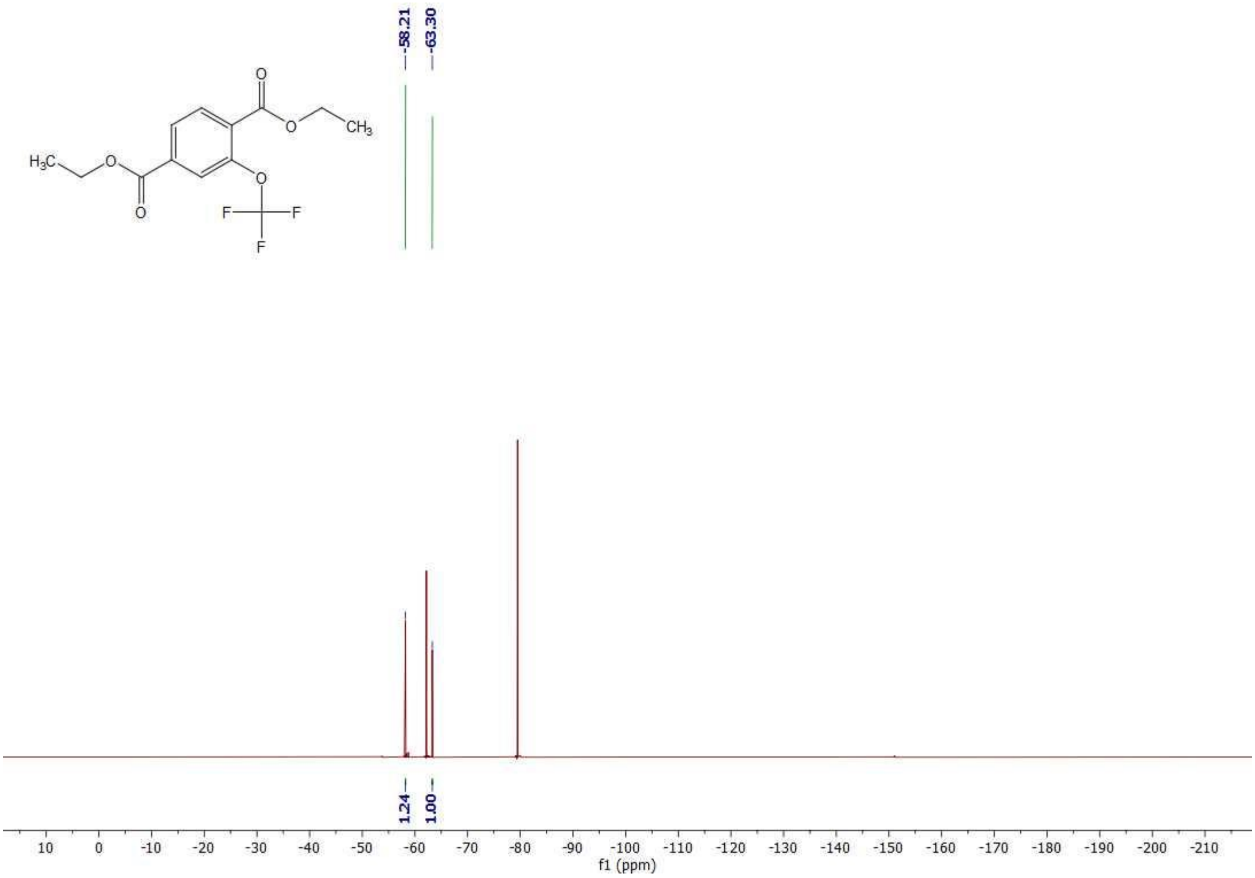

^{19}F NMR spectrum of **18**.

Methyl 4-cyano-2-(trifluoromethoxy)benzoate (19).

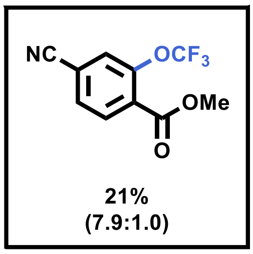

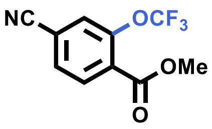

Methyl 4-cyano-2-(trifluoromethoxy)benzoate
major

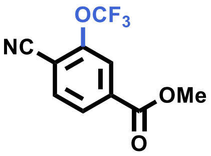

Methyl 4-cyano-3-(trifluoromethoxy)benzoate
minor

The reaction was carried out using methyl 4-cyanobenzoate (152.2 mg, 0.944 mmol) as starting material according to the General procedure 1.

^{19}F NMR yield: 21% (7.9 : 1.0).

^{19}F NMR major isomer (376 MHz, $CDCl_3/CH_3CN$): δ (ppm) = -58.68 (d, *J* = 1.2 Hz, 3F,).

^{19}F NMR minor isomer (376 MHz, $CDCl_3/CH_3CN$): δ (ppm) =-58.40 (s, 3F).

Compounds are known [2].

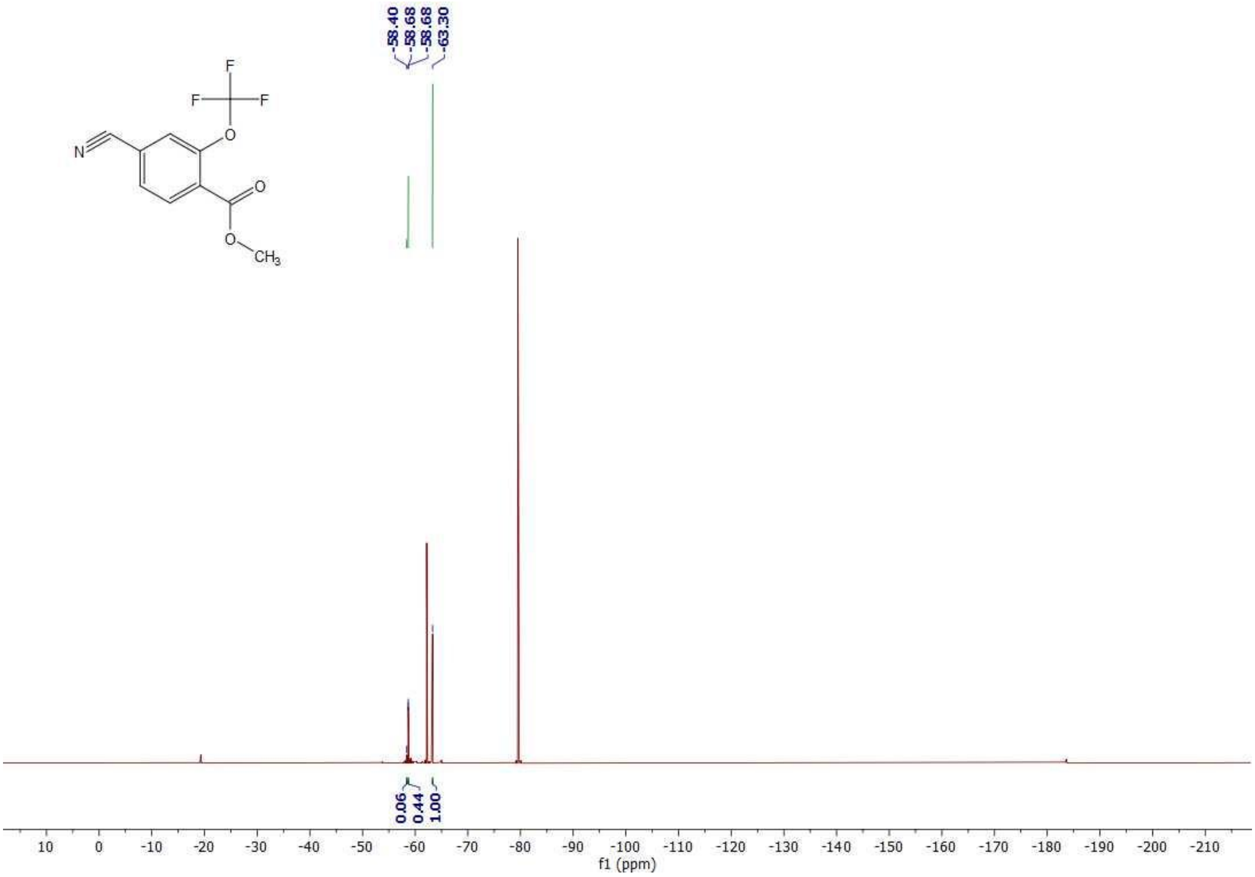

^{19}F NMR spectrum of **19**.

3-(Trifluoromethoxy)benzoic acid (20).

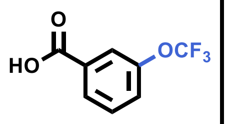

61%
(*o*:*m*:*p* = 1.8:2.4:1.0)

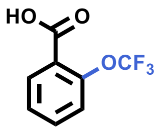

2-(Trifluoromethoxy)benzoic acid
(*o*-isomer)

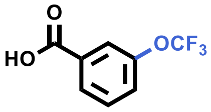

3-(Trifluoromethoxy)benzoic acid
(*m*-isomer)

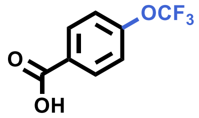

4-(Trifluoromethoxy)benzoic acid
(*p*-isomer)

The reaction was carried out using benzoic acid (115.3 mg, 0.944 mmol) as starting material according to the General procedure 1.

^{19}F NMR yield: 61% (*o* : *m* : *p* = 1.8 : 2.4 : 1.0).

^{19}F NMR *o*-isomer (376 MHz, $CDCl_3/CH_3CN$): δ (ppm) = -58.19 (s, 3F).

^{19}F NMR *m*-isomer (376 MHz, $CDCl_3/CH_3CN$): δ (ppm) = -58.71 (s, 3F).

^{19}F NMR *p*-isomer (376 MHz, $CDCl_3/CH_3CN$): δ (ppm) = -58.42 (s, 3F).

Compounds are known [1,2].

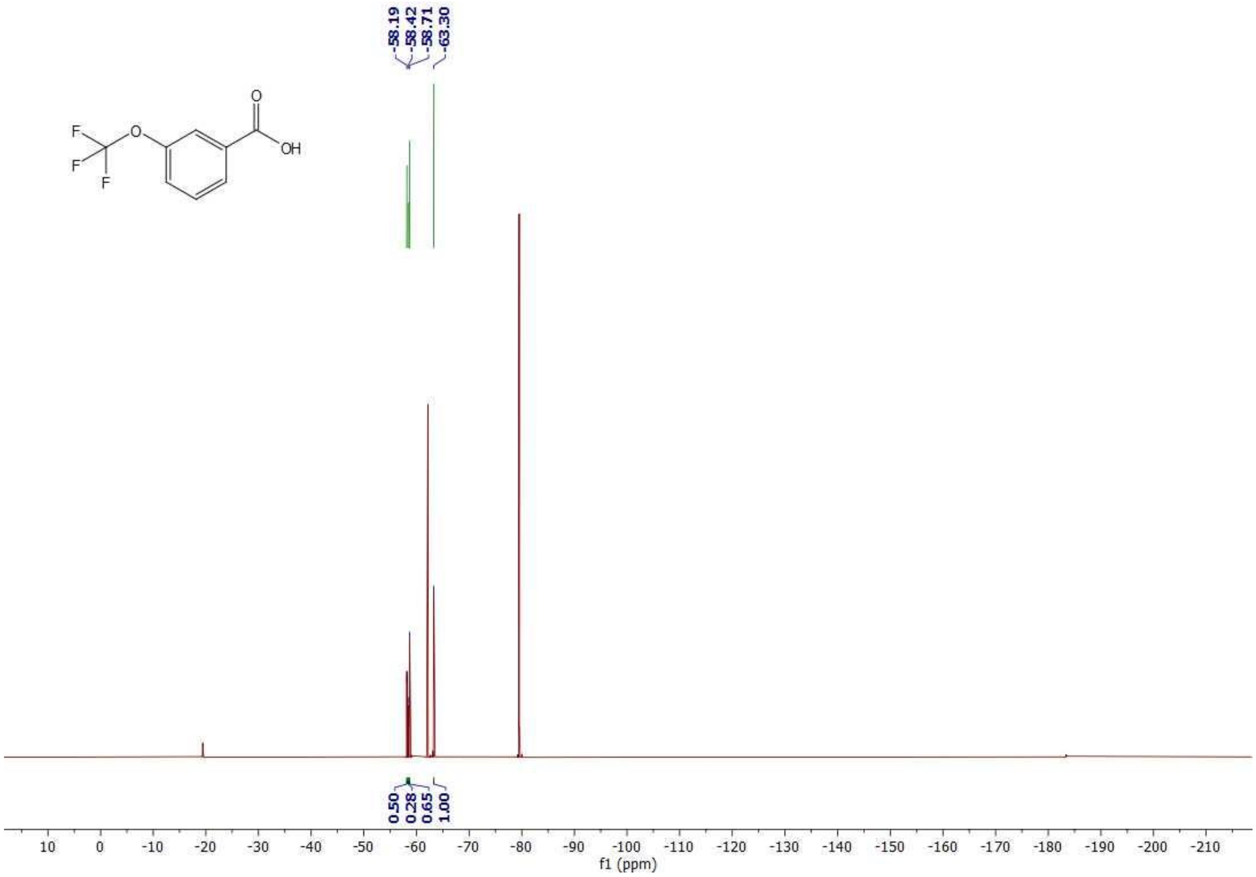

^{19}F NMR spectrum of **20**.

3,5-Difluoro-2-(trifluoromethoxy)benzoic acid (21).

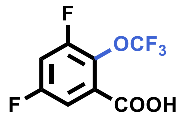

55%
(3.1:1.0)

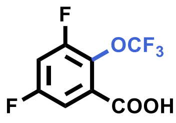

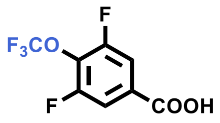

3,5-Difluoro-2-(trifluoromethoxy)benzoic acid
major

3,5-Difluoro-4-(trifluoromethoxy)benzoic acid
minor

The reaction was carried out using 3,5-difluorobenzoic acid (149.2 mg, 0.944 mmol) as starting material according to the General procedure 1.

^{19}F NMR yield: 55% (3.1 : 1.0).

^{19}F NMR major isomer (376 MHz, $CDCl_3/CH_3CN$): δ (ppm) = -59.44 (d, J = 9.3 Hz, 3F), -109.45 – -109.58 (m, 1F), -122.43 – -122.59 (m, 1F).

^{19}F NMR minor isomer (376 MHz, $CDCl_3/CH_3CN$): δ (ppm) = -60.24 (t, J = 7.5 Hz, 3F), -124.63 – -124.74 (m, 2F).

Compounds are known [1].

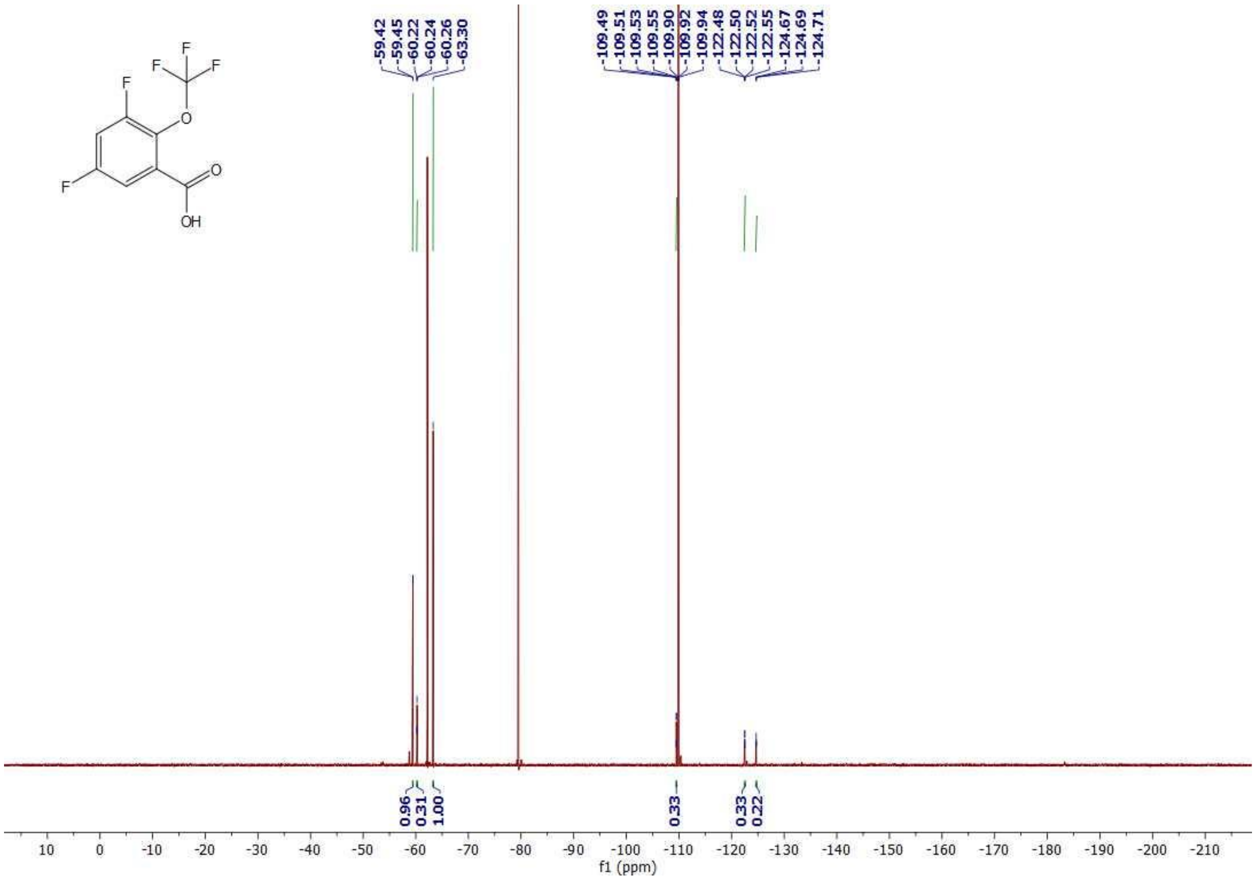

^{19}F NMR spectrum of **21**.

3-(Trifluoromethoxy)benzamide (22).

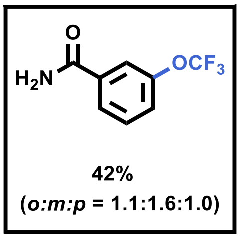

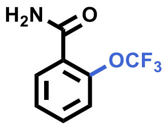

2-(Trifluoromethoxy)benzamide (*o*-isomer)

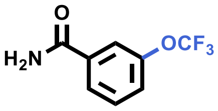

3-(Trifluoromethoxy)benzamide (*m*-isomer)

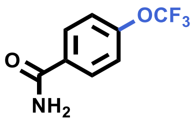

4-(Trifluoromethoxy)benzamide (*p*-isomer)

The reaction was carried out using benzamide (114.3 mg, 0.944 mmol) as starting material according to the General procedure 1.

^{19}F NMR yield: 42% (*o* : *m* : *p* = 1.1 : 1.6 : 1.0).

^{19}F NMR *o*-isomer (376 MHz, $CDCl_3/CH_3CN$): δ (ppm) = -58.19 (d, *J* = 1.4 Hz, 3F).

^{19}F NMR *m*-isomer (376 MHz, $CDCl_3/CH_3CN$): δ (ppm) = -58.60 (s, 3F).

^{19}F NMR *p*-isomer (376 MHz, $CDCl_3/CH_3CN$): δ (ppm) = -58.44 (s, 3F).

Compounds are known [2].

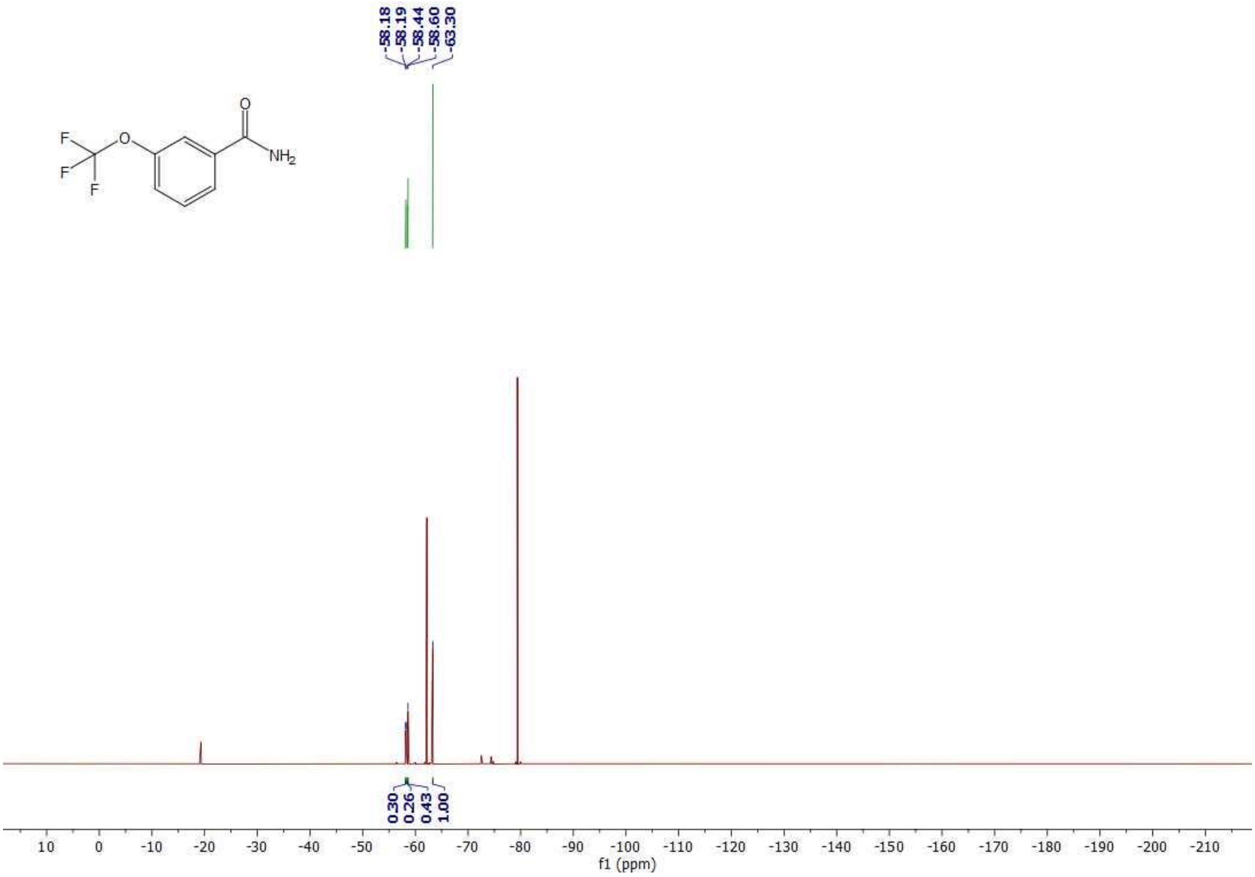

^{19}F NMR spectrum of **22**.

2-Methyl-4-(trifluoromethoxy)isoindoline-1,3-dione (23).

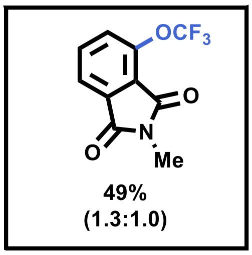

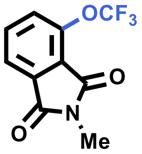

2-Methyl-4-(trifluoromethoxy)isoindoline-1,3-dione
major

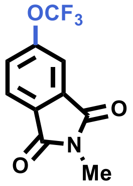

2-Methyl-5-(trifluoromethoxy)isoindoline-1,3-dione
minor

The reaction was carried out using 2-methylisoindoline-1,3-dione (152.2 mg, 0.944 mmol) as starting material according to the General procedure 1.

^{19}F NMR yield: 49% (1.3 : 1.0).

^{19}F NMR major isomer (376 MHz, $CDCl_3/CH_3CN$): δ (ppm) = -58.61 (s, 3F).

^{19}F NMR minor isomer (376 MHz, $CDCl_3/CH_3CN$): δ (ppm) =-58.64 (s, 3F).

Compounds are known [2].

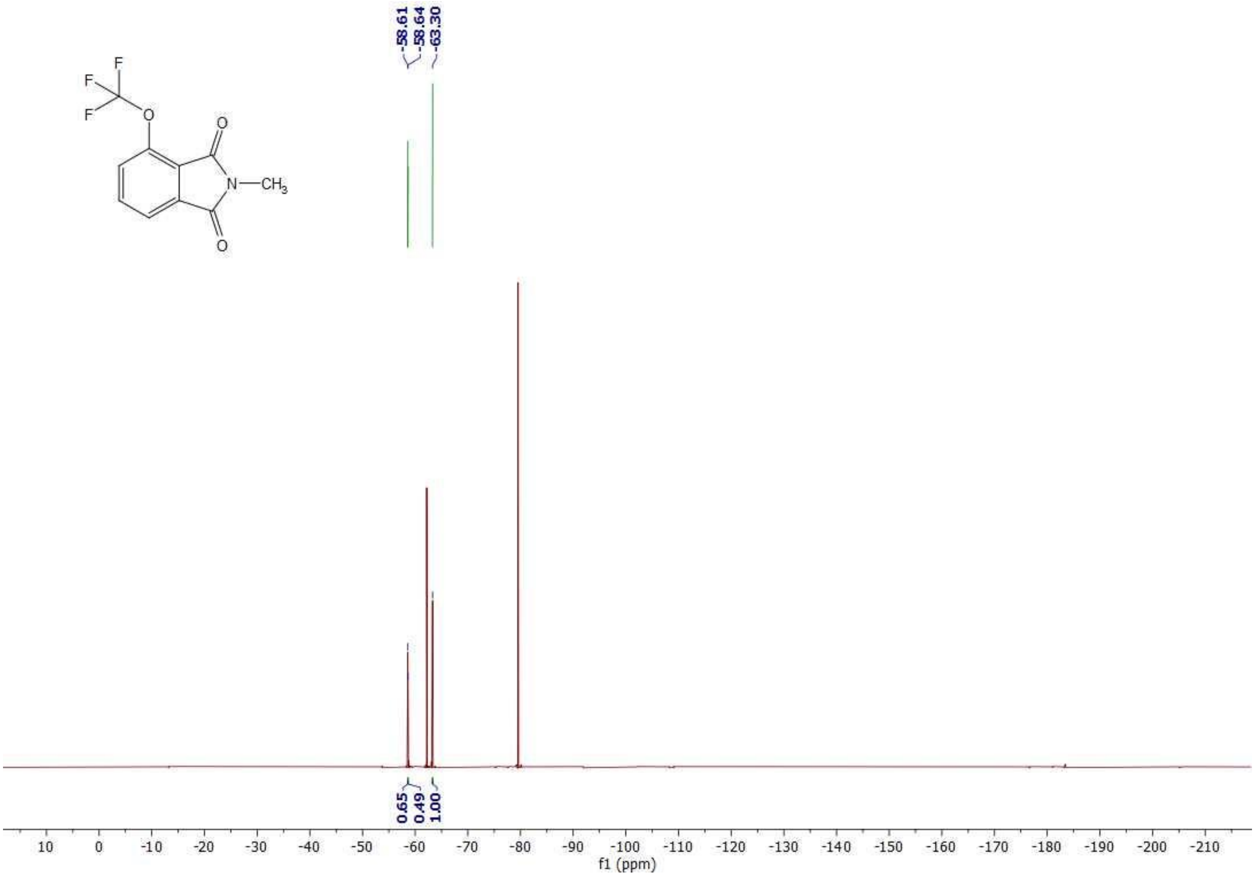

^{19}F NMR spectrum of **23**.

2-(Trifluoromethoxy)benzonitrile (24).

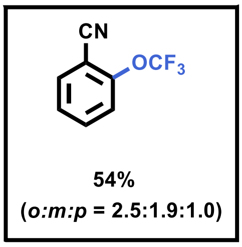

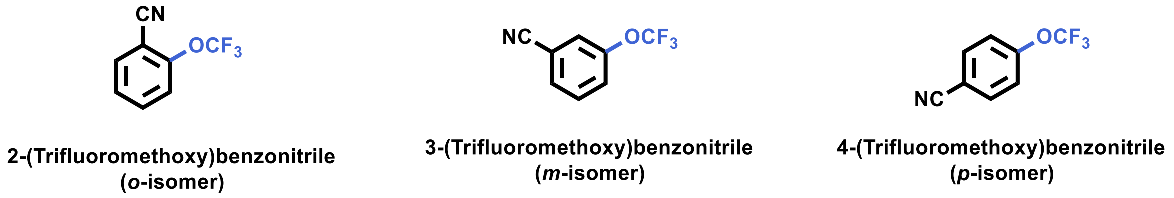

The reaction was carried out using benzonitrile (97.3 mg, 0.944 mmol) as starting material according to the General procedure 1.

^{19}F NMR yield: 54% (*o* : *m* : *p* = 2.5 : 1.9 : 1.0).

^{19}F NMR *o*-isomer (376 MHz, $CDCl_3/CH_3CN$): δ (ppm) = -58.68 (d, *J* = 1.8 Hz, 3F).

^{19}F NMR *m*-isomer (376 MHz, $CDCl_3/CH_3CN$): δ (ppm) =-58.80 (s, 3F).

^{19}F NMR *p*-isomer (376 MHz, $CDCl_3/CH_3CN$): δ (ppm) = -58.48 (s, 3F).

Compounds are known [2].

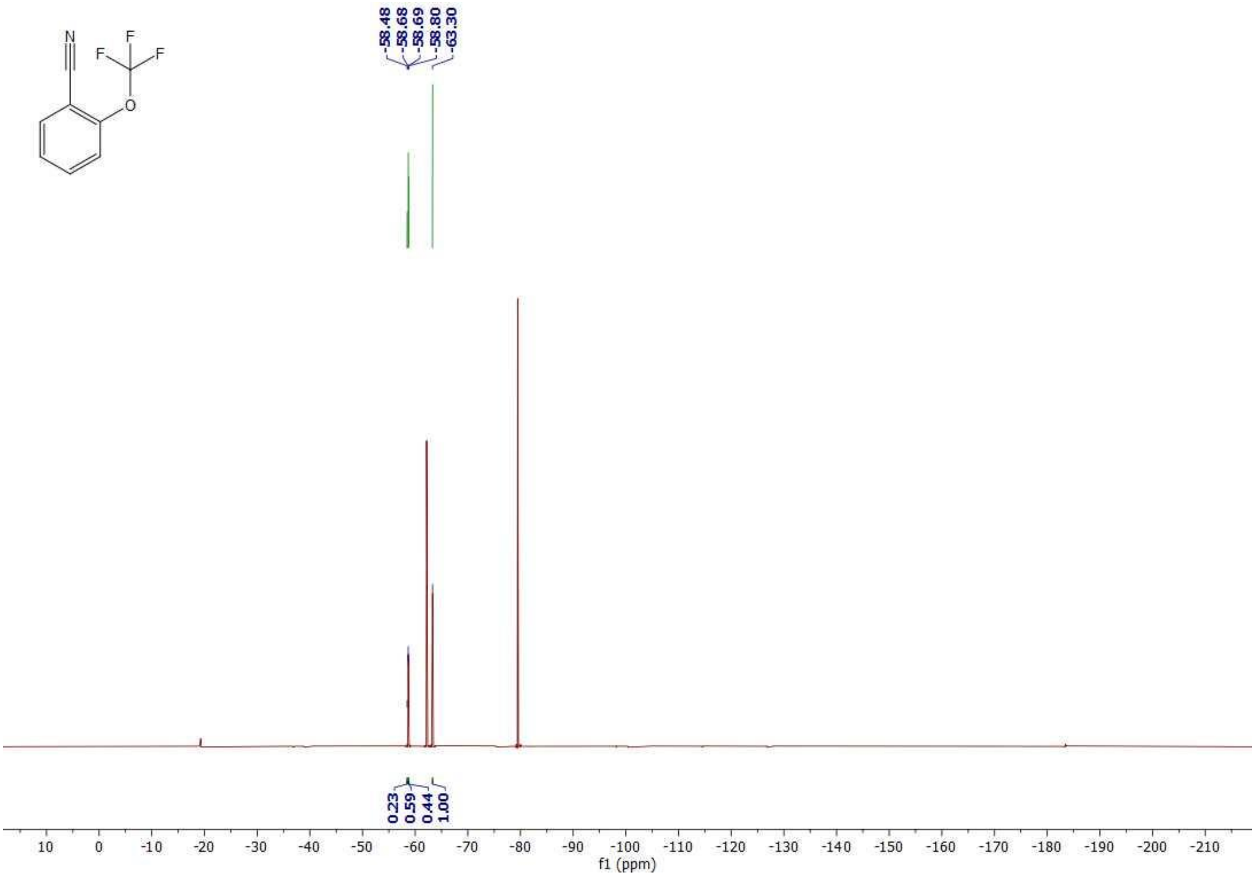

^{19}F NMR spectrum of **24**.

2-(Trifluoromethoxy)-[1,1'-biphenyl]-4,4'-dicarbonitrile (25).

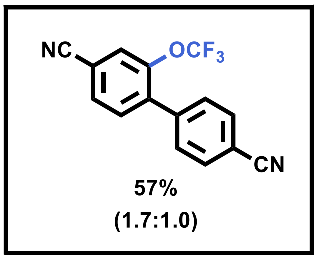

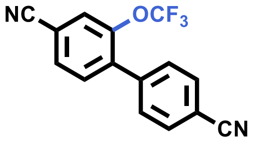

2-(Trifluoromethoxy)-[1,1'-biphenyl]-4,4'-dicarbonitrile
major

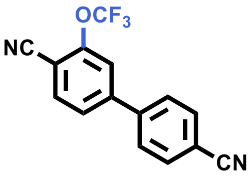

3-(Trifluoromethoxy)-[1,1'-biphenyl]-4,4'-dicarbonitrile
minor

The reaction was carried out using [1,1'-biphenyl]-4,4'-dicarbonitrile (192.8 mg, 0.944 mmol) as starting material according to the General procedure 1.

^{19}F NMR yield: 57% (1.7:1.0).

^{19}F NMR major isomer (376 MHz, $CDCl_3/CH_3CN$): δ (ppm) = -58.27 (s, 3F).

^{19}F NMR minor isomer (376 MHz, $CDCl_3/CH_3CN$): δ (ppm) = -58.47 (s, 3F).

Compounds are known [2].

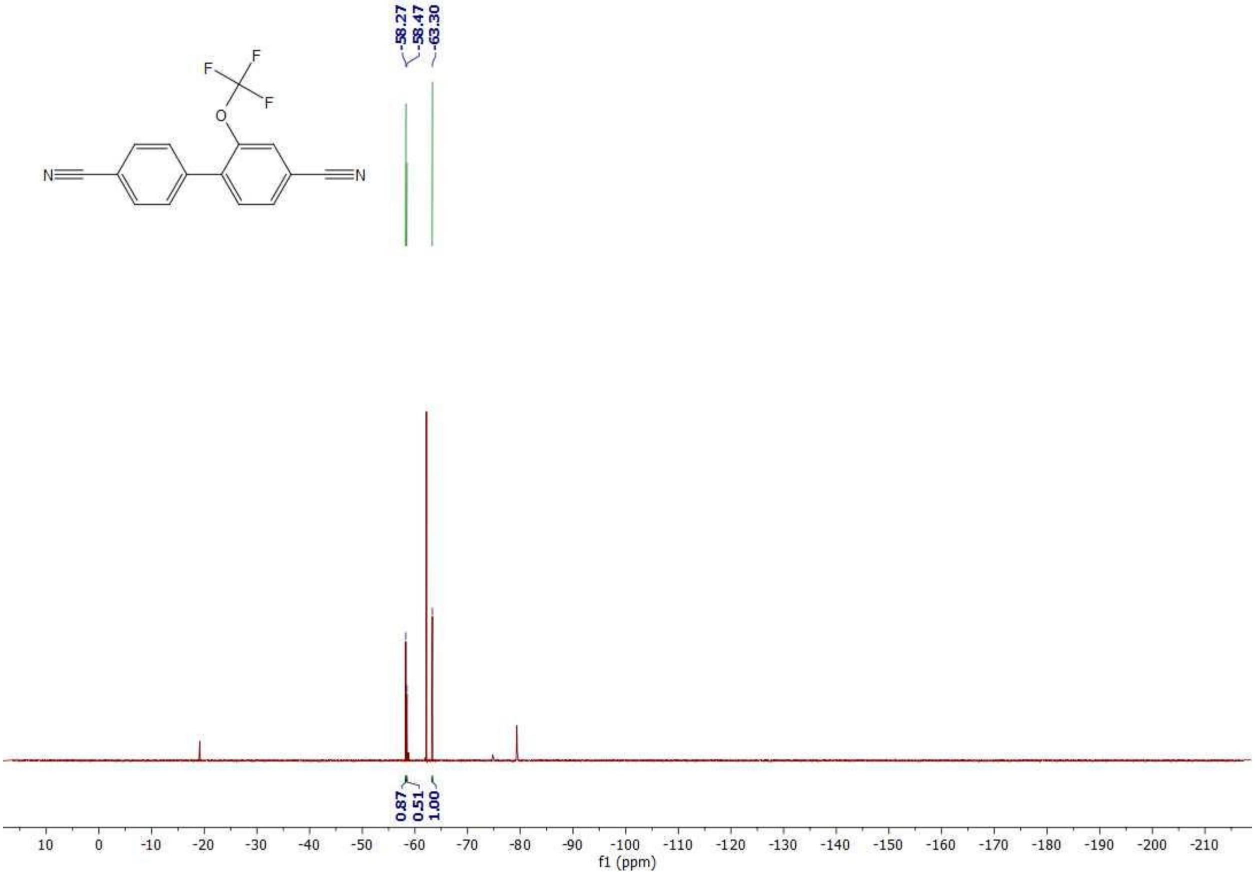

^{19}F NMR spectrum of **25**.

1-Methyl-2-(trifluoromethoxy)benzene (26).

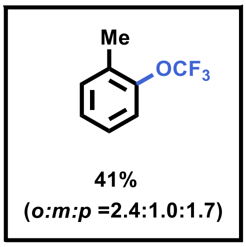

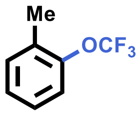

1-Methyl-2-(trifluoromethoxy)benzene (*o*-isomer)

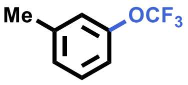

1-Methyl-3-(trifluoromethoxy)benzene (*m*-isomer)

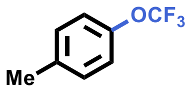

1-Methyl-4-(trifluoromethoxy)benzene (*p*-isomer)

The reaction was carried out using toluene (86.9 mg, 0.944 mmol) as starting material according to the General procedure 1.

^{19}F NMR yield: 41% (*o* : *m* : *p* = 2.4 : 1.0 : 1.7).

^{19}F NMR *o*-isomer (376 MHz, $CDCl_3/CH_3CN$): δ (ppm) = -58.13 (s, 3F).

^{19}F NMR *m*-isomer (376 MHz, $CDCl_3/CH_3CN$): δ (ppm) =-58.42 (s, 3F).

^{19}F NMR *p*-isomer (376 MHz, $CDCl_3/CH_3CN$): δ (ppm) = -58.69 (s, 3F).

Compounds are known [1].

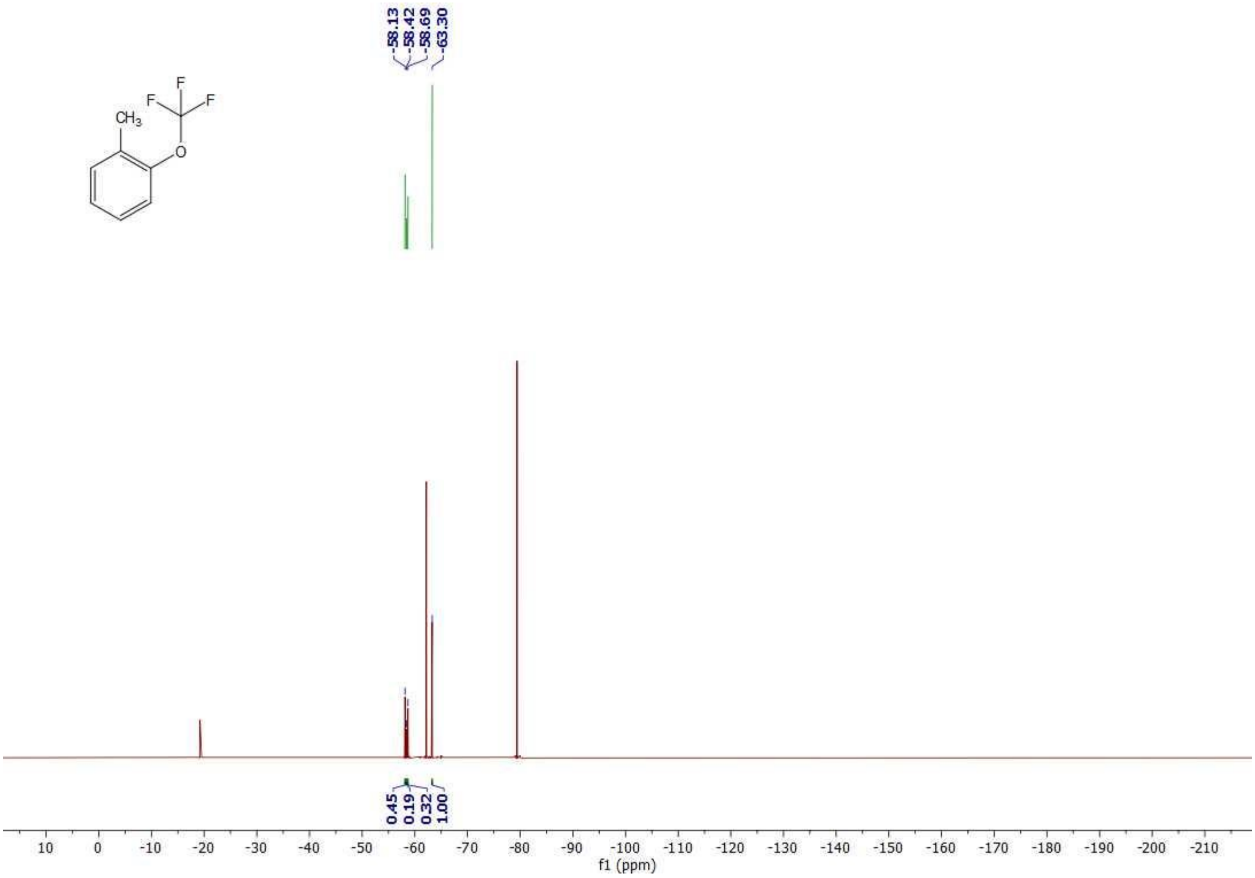

^{19}F NMR spectrum of **26**.

1-(*tert*-Butyl)-4-(trifluoromethoxy)benzene (27).

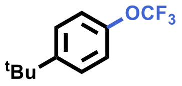

51%
(*o*:*m*:*p* =1.0:2.6:3.6)

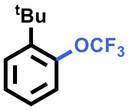

1-(*tert*-Butyl)-2-(trifluoromethoxy)benzene (*o*-isomer)

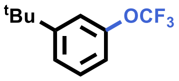

1-(*tert*-Butyl)-3-(trifluoromethoxy)benzene (*m*-isomer)

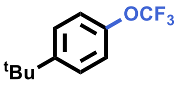

1-(*tert*-Butyl)-4-(trifluoromethoxy)benzene (*p*-isomer)

The reaction was carried out using *tert*-butylbenzene (126.7 mg, 0.944 mmol) as starting material according to the General procedure 1.

^{19}F NMR yield: 51% (*o* : *m* : *p* = 1.0 : 2.6 : 3.6).

^{19}F NMR *o*-isomer (376 MHz, $CDCl_3/CH_3CN$): δ (ppm) = -55.16 (s, 3F).

^{19}F NMR *m*-isomer (376 MHz, $CDCl_3/CH_3CN$): δ (ppm) = -58.43 (s, 3F).

^{19}F NMR *p*-isomer (376 MHz, $CDCl_3/CH_3CN$): δ (ppm) = -58.63 (s, 3F).

Compounds are known [1].

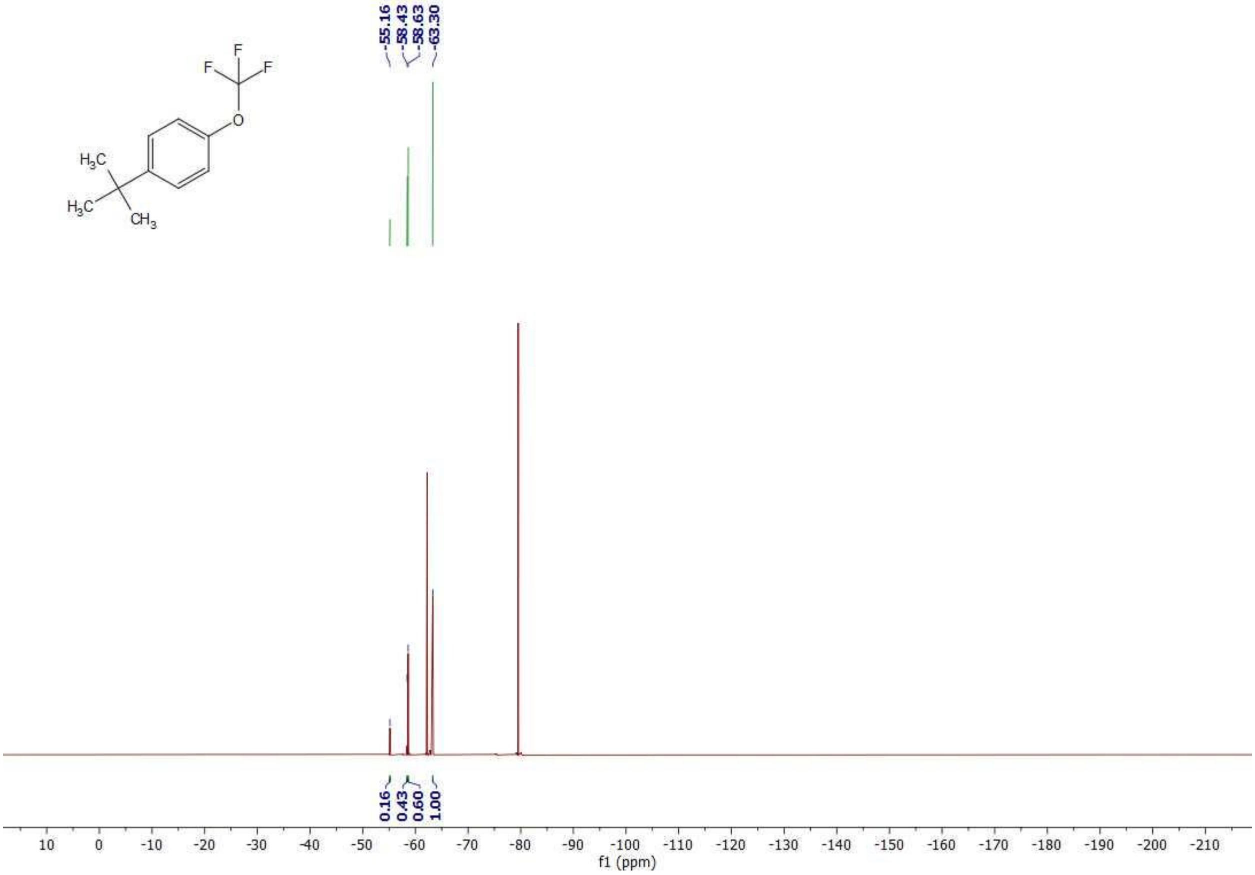

^{19}F NMR spectrum of **27**.

1-Nitro-3-(trifluoromethoxy)benzene (28).

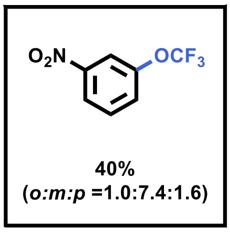

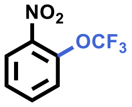

1-Nitro-2-(trifluoromethoxy)benzene (*o*-isomer)

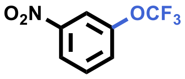

1-Nitro-3-(trifluoromethoxy)benzene (*m*-isomer)

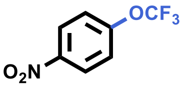

1-Nitro-4-(trifluoromethoxy)benzene (*p*-isomer)

The reaction was carried out using nitrobenzene (116.2 mg, 0.944 mmol) as starting material according to the General procedure 1.

^{19}F NMR yield: 40% (*o* : *m* : *p* = 1.0 : 7.4 : 1.6).

^{19}F NMR *o*-isomer (376 MHz, $CDCl_3/CH_3CN$): δ (ppm) = -58.45 (s, 3F).

^{19}F NMR *m*-isomer (376 MHz, $CDCl_3/CH_3CN$): δ (ppm) =-58.85 (s, 3F).

^{19}F NMR *p*-isomer (376 MHz, $CDCl_3/CH_3CN$): δ (ppm) =-58.51 (s, 3F).

Compounds are known [2].

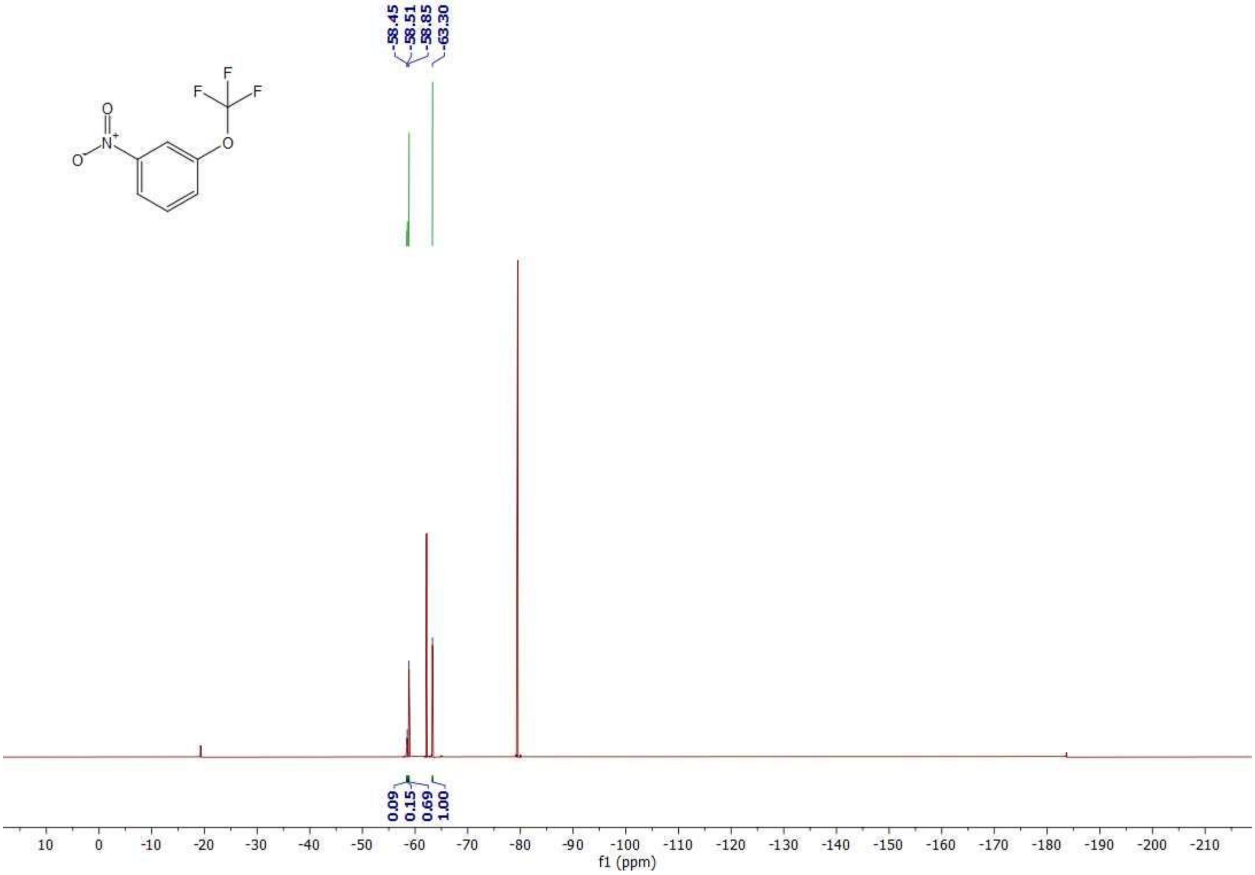

^{19}F NMR spectrum of **28**.

1-(Phenylsulfonyl)-3-(trifluoromethoxy)benzene (29).

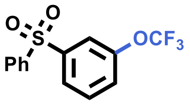

65%
(*o*:*m*:*p* =1.0:3.5:1.2)

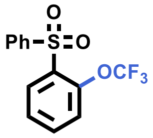

1-(Phenylsulfonyl)-2-
(trifluoromethoxy)benzene
(*o*-isomer)

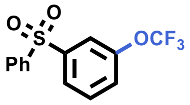

1-(Phenylsulfonyl)-3-
(trifluoromethoxy)benzene
(*m*-isomer)

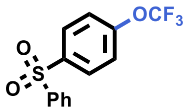

1-(Phenylsulfonyl)-4-
(trifluoromethoxy)benzene
(*p*-isomer)

The reaction was carried out using sulfonyldibenzene (206.1 mg, 0.944 mmol) as starting material according to the General procedure 1.

^{19}F NMR yield: 65% (*o* : *m* : *p* = 1.0 : 3.5 : 1.2).

^{19}F NMR *o*-isomer (376 MHz, $CDCl_3/CH_3CN$): δ (ppm) = -57.09 (d, *J* = 1.9 Hz, 3F).

^{19}F NMR *m*-isomer (376 MHz, $CDCl_3/CH_3CN$): δ (ppm) = -58.73 (s, 3F).

^{19}F NMR *p*-isomer (376 MHz, $CDCl_3/CH_3CN$): δ (ppm) = -58.45 (s, 3F).

Compounds are known [1].

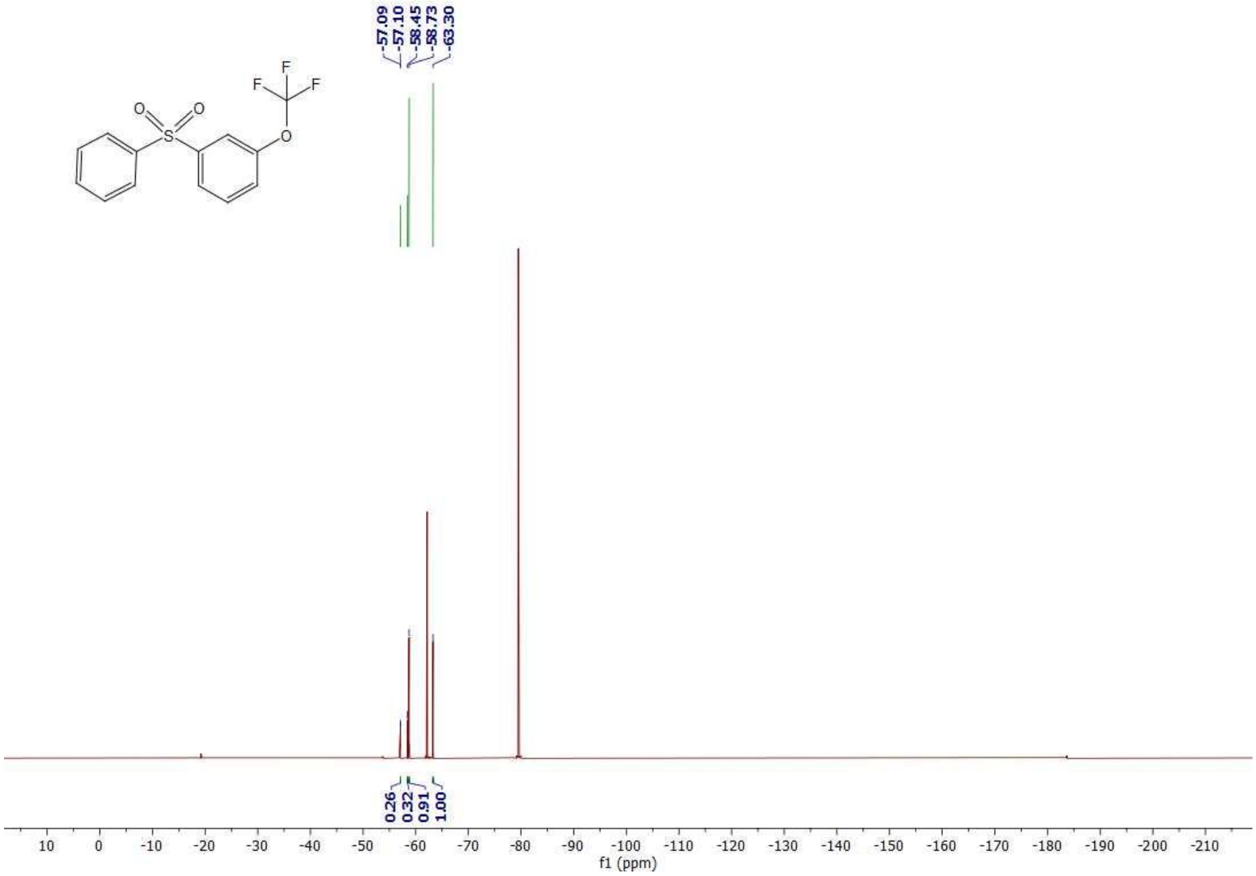

^{19}F NMR spectrum of **29**.

2,2'-(2-(Trifluoromethoxy)-1,4-phenylene)bis(4,4,5,5-tetramethyl-1,3,2-dioxaborolane) (30).

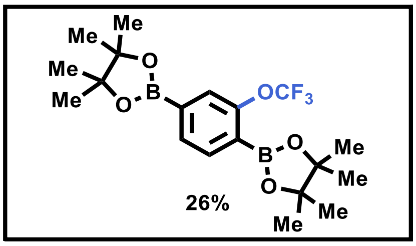

The reaction was carried out using 1,4-bis(4,4,5,5-tetramethyl-1,3,2-dioxaborolan-2-yl)benzene (311.5 mg, 0.944 mmol) as starting material according to the General procedure 1.

^{19}F NMR yield: 26%.

^{19}F NMR (376 MHz, $CDCl_3/CH_3CN$): δ (ppm) = -58.08 (s, 3F).

Compound is known [2].

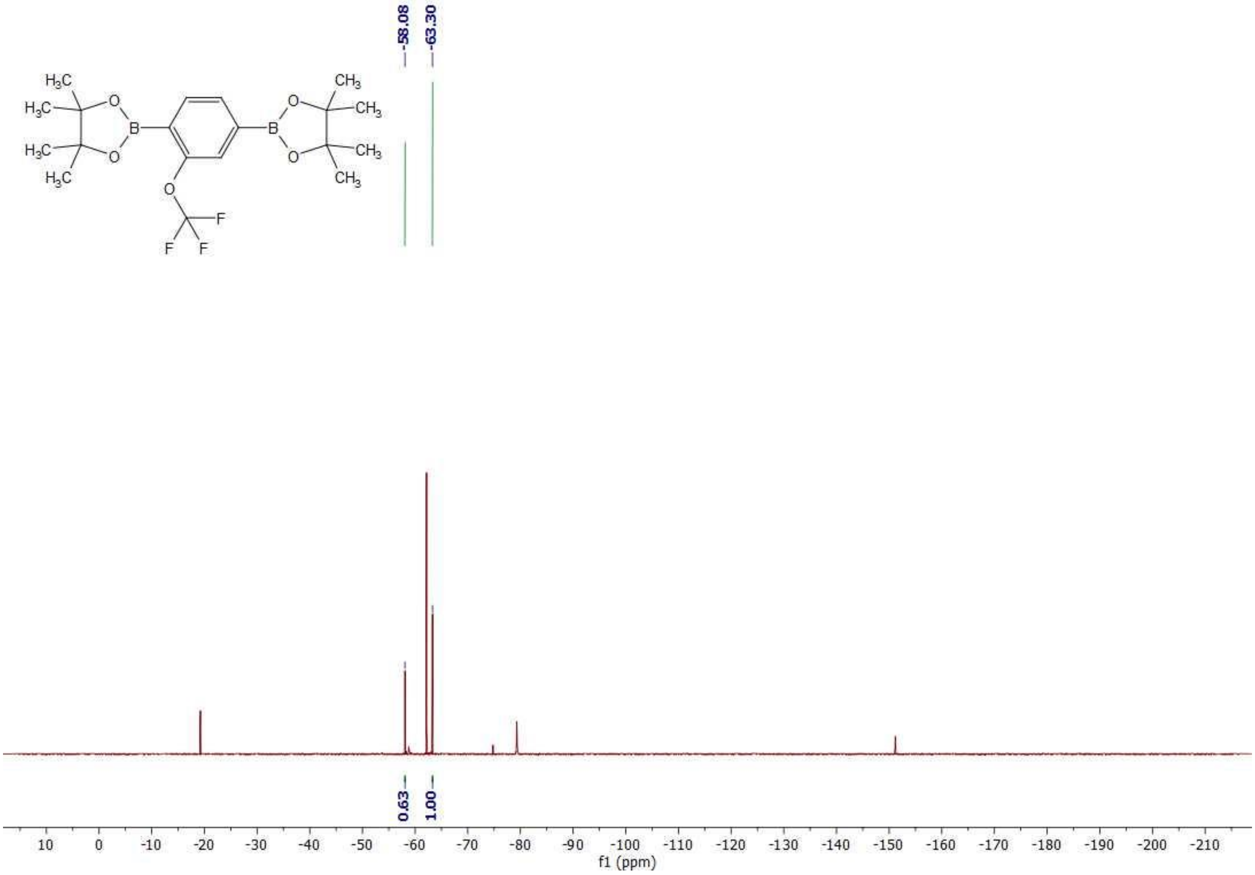

^{19}F NMR spectrum of **30**.

2,6-Dichloro-3-(trifluoromethoxy)pyridine (31).

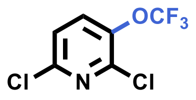

64%

The reaction was carried out using 2,6-dichloropyridine (139.7 mg, 0.944 mmol) as starting material according to the General procedure 1.

^{19}F NMR yield: 64%.

^{19}F NMR (376 MHz, $CDCl_3/CH_3CN$): δ (ppm) = -58.93 (s, 3F).

Compound is known [1].

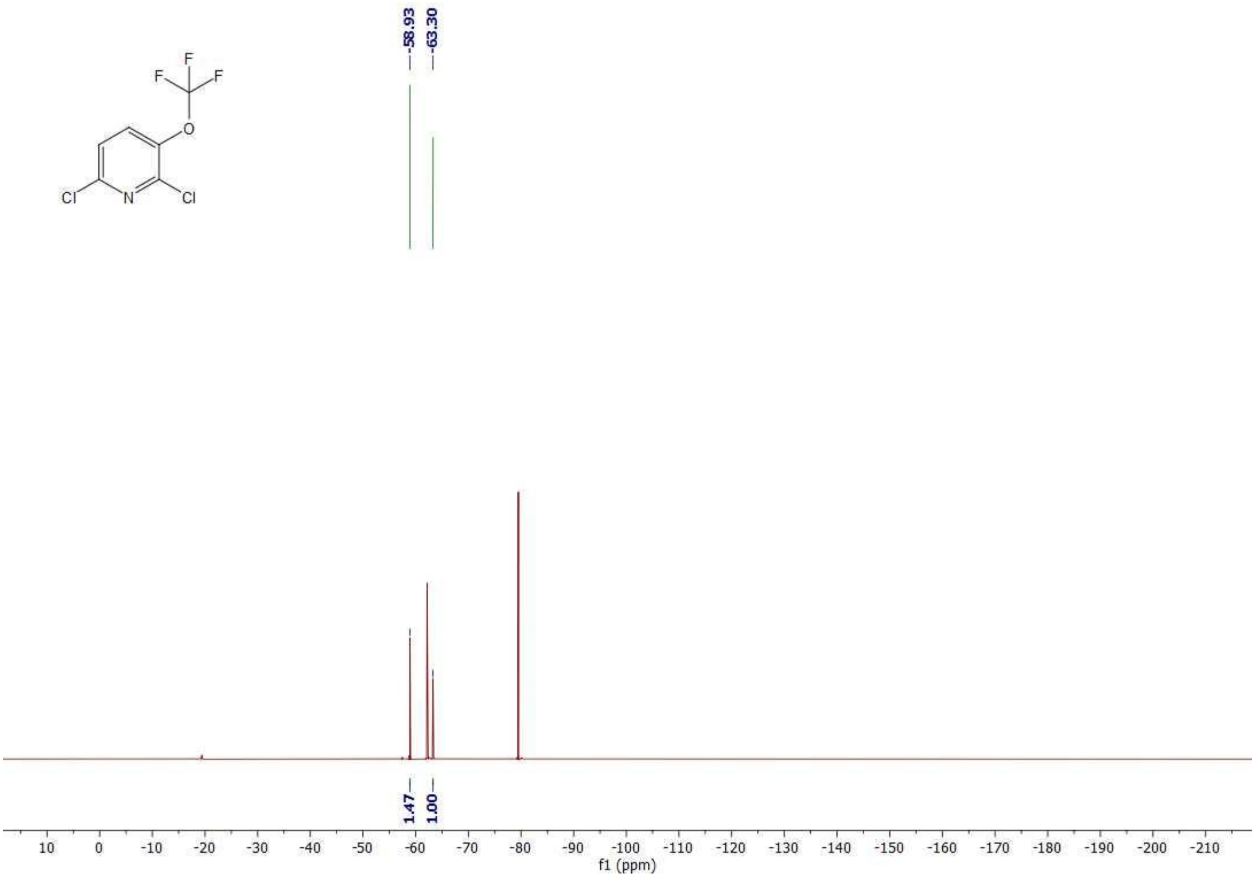

^{19}F NMR spectrum of **31**.

2,4,6-Trichloro-5-(trifluoromethoxy)pyrimidine (32).

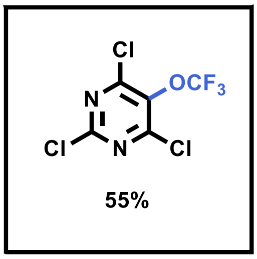

The reaction was carried out using 2,4,6-trichloropyrimidine (173.1 mg, 0.944 mmol) as starting material according to the General procedure 1.

^{19}F NMR yield: 55%.

^{19}F NMR (376 MHz, $CDCl_3/CH_3CN$): δ (ppm) = -57.11 (s, 3F).

Compound is known [1].

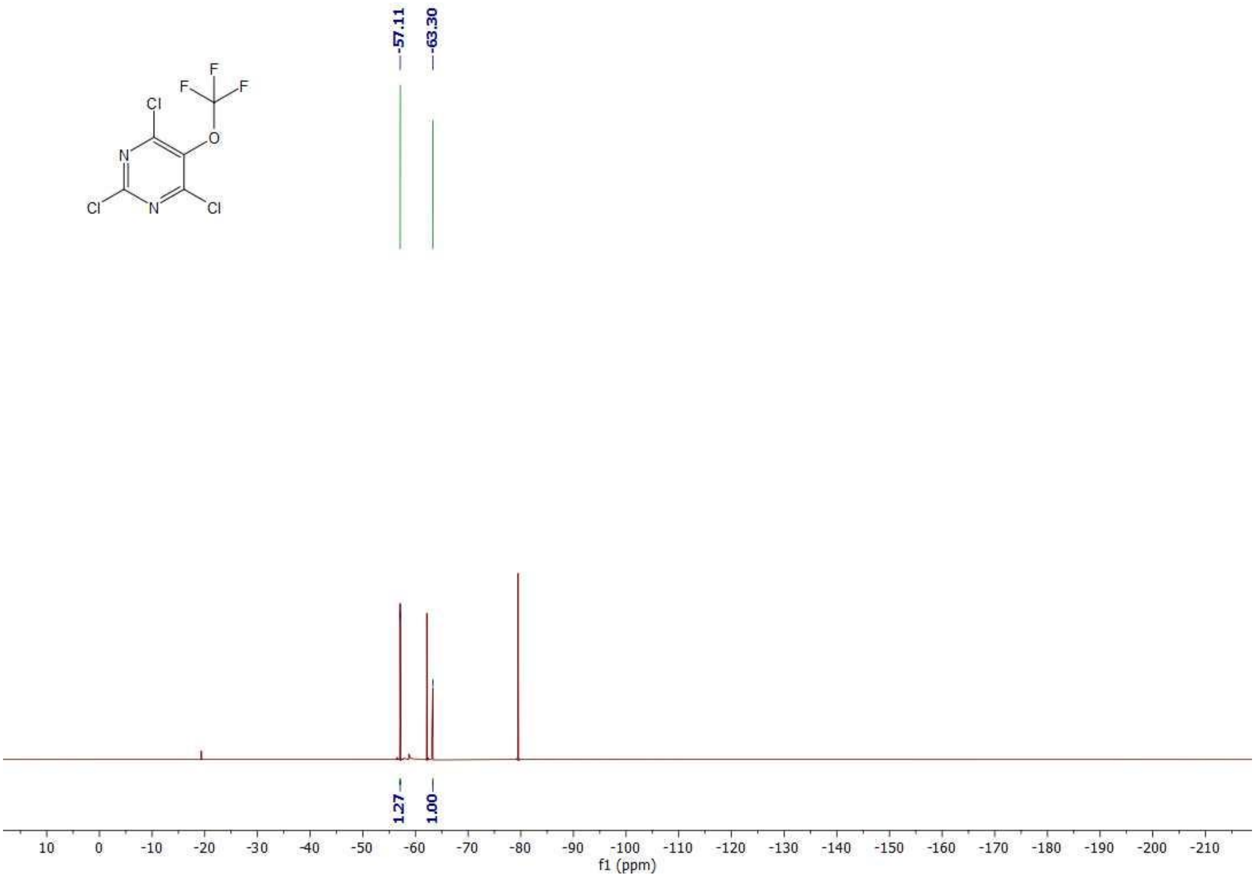

^{19}F NMR spectrum of **32**.

4,6-Dichloro-2-methyl-5-(trifluoromethoxy)pyrimidine (33).

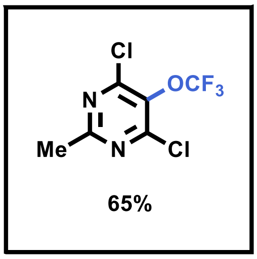

The reaction was carried out using 4,6-dichloro-2-methylpyrimidine (153.9 mg, 0.944 mmol) as starting material according to the General procedure 1.

^{19}F NMR yield: 65%.

^{19}F NMR (376 MHz, $CDCl_3/CH_3CN$): δ (ppm) = -57.32 (s, 3F).

Compound is known [1].

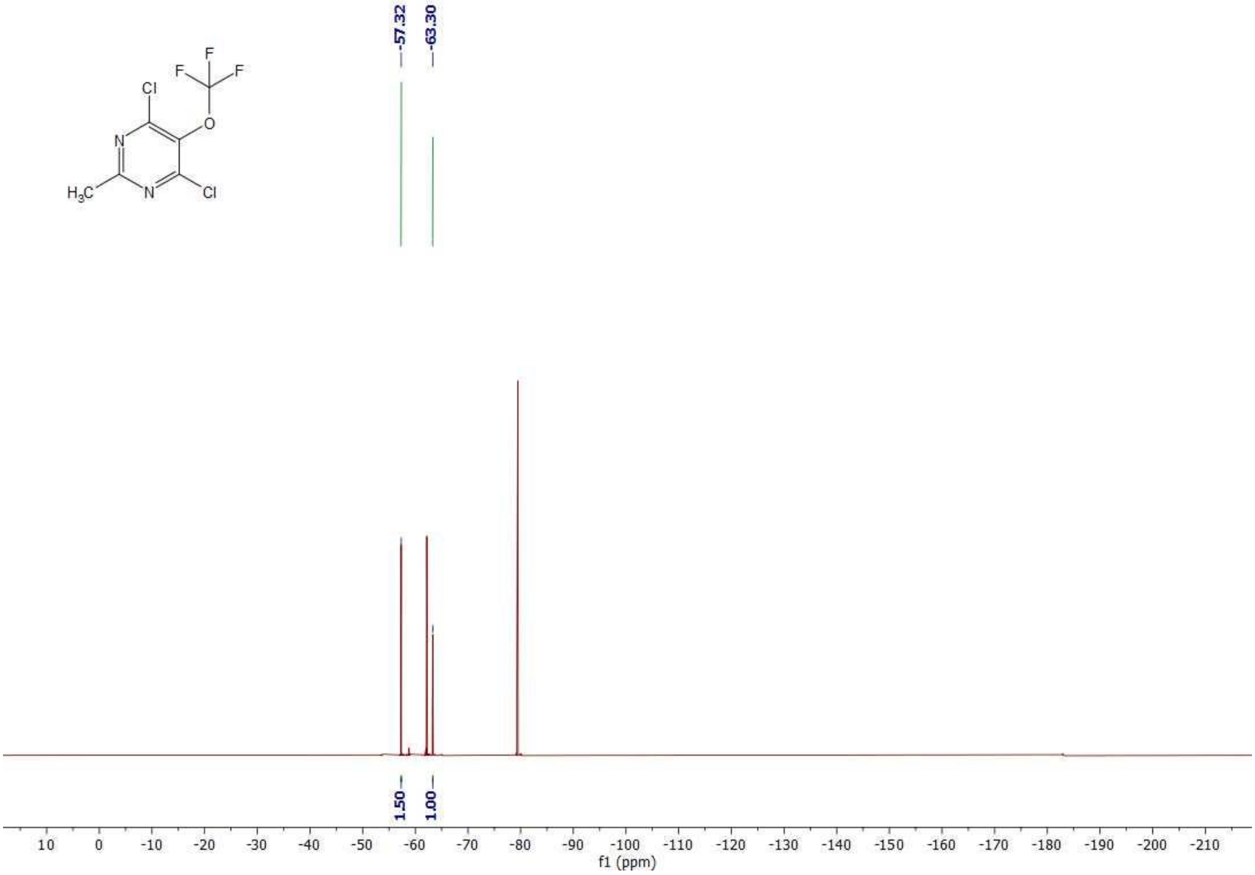

^{19}F NMR spectrum of **33**.

4-Bromo-5-(trifluoromethoxy)thiophene-2-carbonitrile (34).

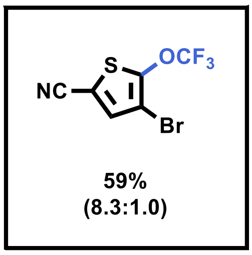

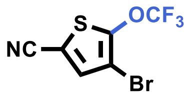

4-Bromo-5-(trifluoromethoxy)thiophene-2-carbonitrile
major

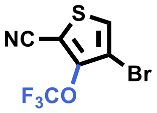

4-Bromo-3-(trifluoromethoxy)thiophene-2-carbonitrile
minor

The reaction was carried out using 4-bromothiophene-2-carbonitrile (177.5 mg, 0.944 mmol) as starting material according to the General procedure 1.

^{19}F NMR yield: 59% (8.3 : 1.0).

^{19}F NMR major isomer (376 MHz, $CDCl_3/CH_3CN$): δ (ppm) = -60.62 (s, 3F).

^{19}F NMR minor isomer (376 MHz, $CDCl_3/CH_3CN$): δ (ppm) =-58.38 (s, 3F).

Compounds are known [1].

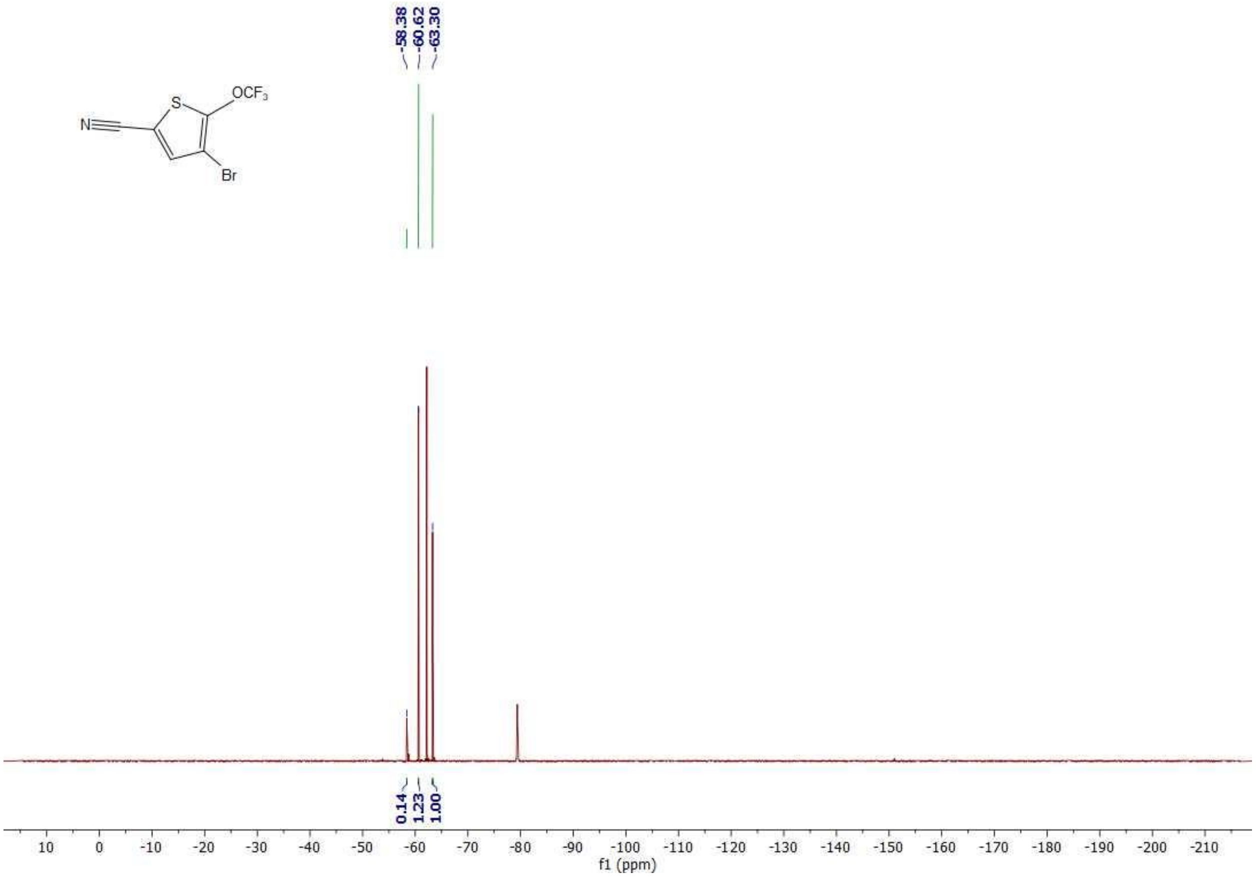

^{19}F NMR spectrum of **34**.

4-Bromo-5-(trifluoromethoxy)thiophene-2-carboxylic acid (35).

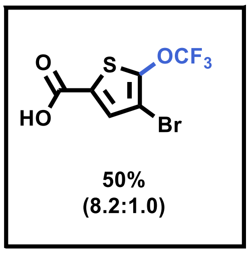

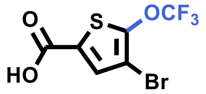

4-Bromo-5-(trifluoromethoxy)thiophene-2-carboxylic acid major

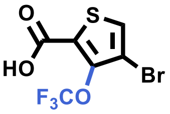

4-Bromo-3-(trifluoromethoxy)thiophene-2-carboxylic acid minor

The reaction was carried out using 4-bromothiophene-2-carboxylic acid (195.5 mg, 0.944 mmol) as starting material according to the General procedure 1.

^{19}F NMR yield: 50% (8.2 : 1.0).

^{19}F NMR major isomer (376 MHz, $CDCl_3/CH_3CN$): δ (ppm) = -60.64 (s, 3F).

^{19}F NMR minor isomer (376 MHz, $CDCl_3/CH_3CN$): δ (ppm) =-57.69 (s, 3F).

Compounds are known [1].

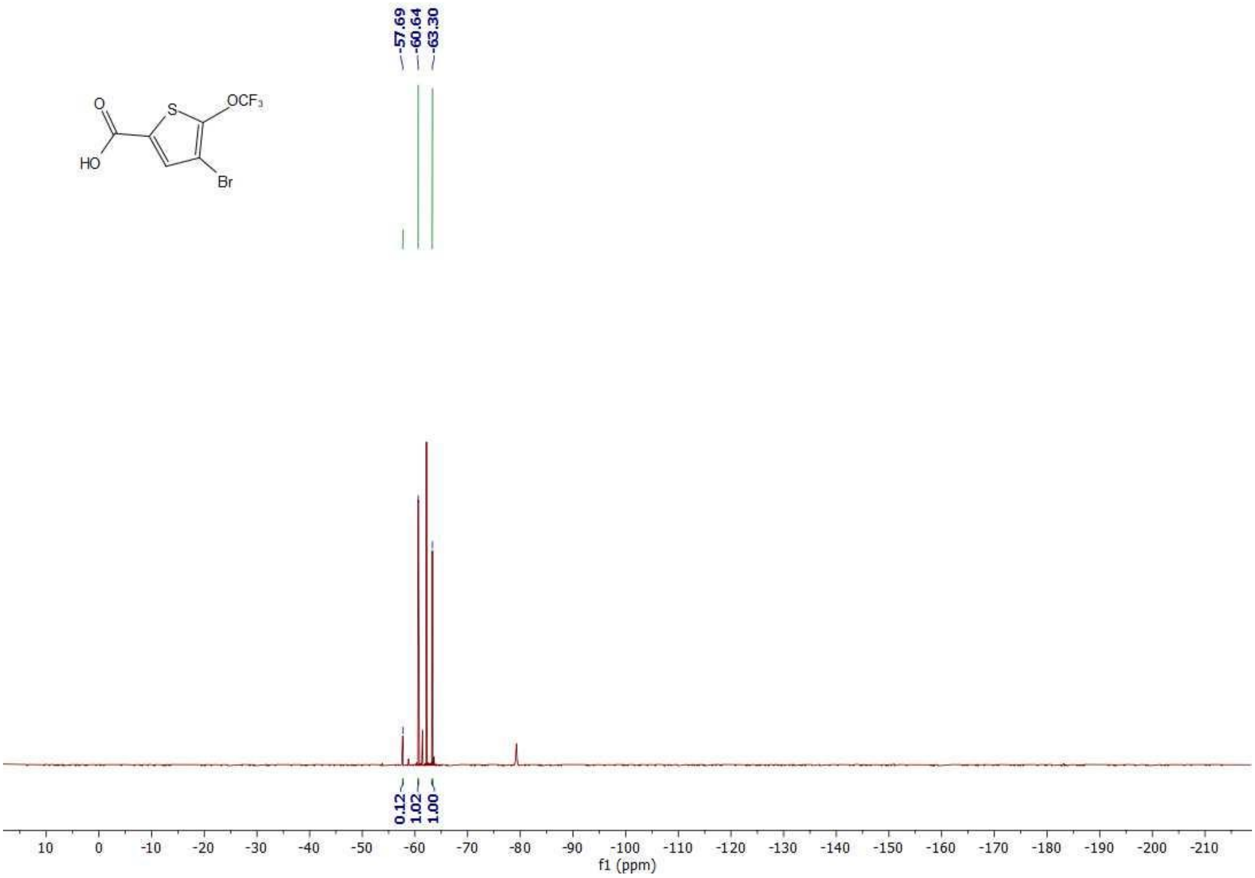

^{19}F NMR spectrum of **35**.

4-Bromo-5-(trifluoromethoxy)thiophene-2-carbaldehyde (36).

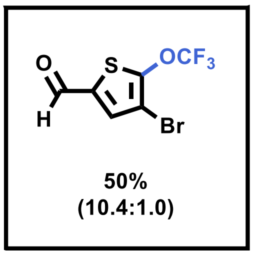

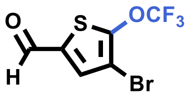

4-Bromo-5-(trifluoromethoxy)thiophene-2-carbaldehyde
major

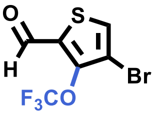

4-Bromo-3-(trifluoromethoxy)thiophene-2-carbaldehyde
minor

The reaction was carried out using 4-bromothiophene-2-carbaldehyde (180.4 mg, 0.944 mmol) as starting material according to the General procedure 1.

^{19}F NMR yield: 50% (10.4 : 1.0).

^{19}F NMR major isomer (376 MHz, $CDCl_3$/CH_3CN): δ (ppm) = -60.49 (s, 3F).

^{19}F NMR minor isomer (376 MHz, $CDCl_3$/CH_3CN): δ (ppm) = -58.76 (s, 3F).

Compounds are known [1].

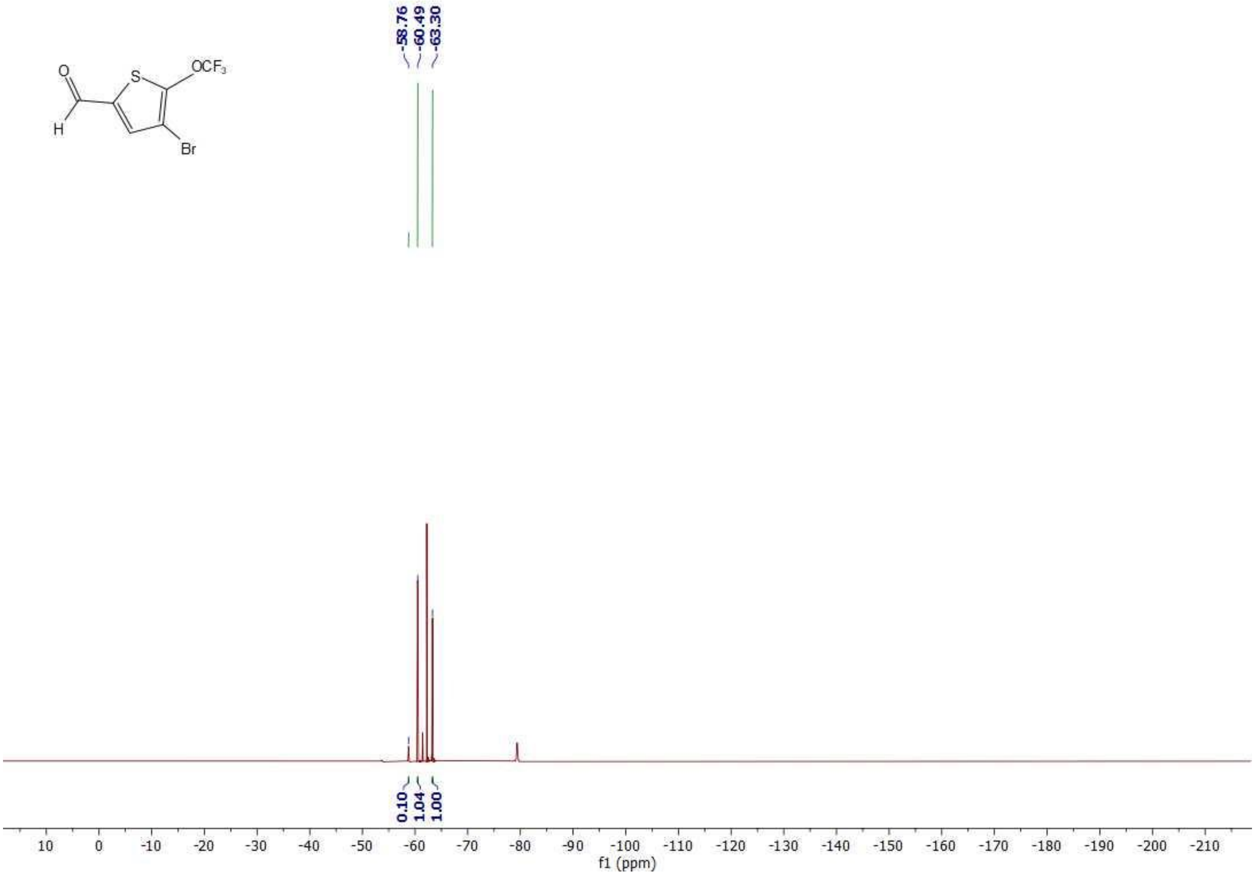

^{19}F NMR spectrum of **36**.

3,4-Dibromo-2-(trifluoromethoxy)thiophene (37).

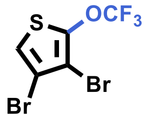

53%

The reaction was carried out using 3,4-dibromothiophene (228.4 mg, 0.944 mmol) as starting material according to the General procedure 1.

^{19}F NMR yield: 53%. ^{19}F NMR (376 MHz, $CDCl_3/CH_3CN$): δ (ppm) = -60.54 (s, 3F).

Compound is known [1].

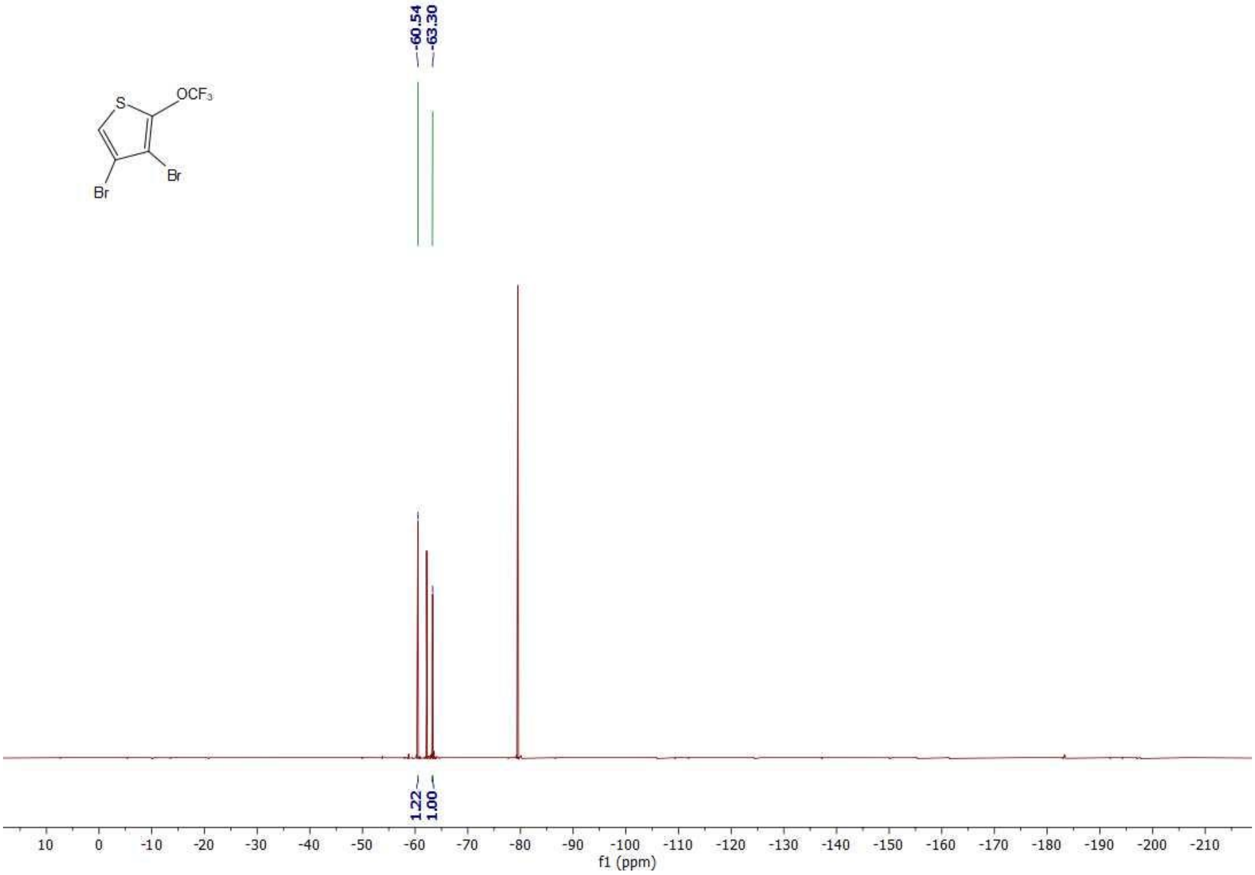

^{19}F NMR spectrum of **37**.
